# Measuring dielectric properties for microwave-assisted extraction of essential oils using single-mode and multimode reactors†

**DOI:** 10.1039/c8ra08727j

**Published:** 2019-02-12

**Authors:** Carlos A. Parizotto, Evandro L. Dall'Oglio, Leonardo G. de Vasconcelos, Paulo T. de Sousa, Eduardo G. R. Taques Filho, Carlos Alberto Kuhnen

**Affiliations:** Departamento de Química, Universidade Federal do Mato Grosso Av. Fernando Corrêa da Costa s/n, Coxipó Cuiabá MT CEP 78090-600 Brazil dalloglio.evandro@gmail.com evandroluiz@ufmt.br +55 65 36158799 +55 65 36158798; Departamento de Física, Universidade Federal do Santa Catarina, Campus Universitário Trindade Florianópolis SC CEP 88040-970 Brazil

## Abstract

The dielectric properties of *Cymbopogon nardus*, *Eucalyptus* sp., *Piper aduncum* and *Piper hispidinervum* were investigated as a function of frequency and temperature, using dry plant matter and its mixtures with water at different concentrations. This was followed by the extraction of essential oils performed with microwave heating in single-mode and multimode cavities with a variable power 6.0 kW generator operating at 2.45 GHz. The dielectric properties of the dry plant matter changed markedly with increasing water content, exhibiting high loss factors and small penetration depths. Due to the high level of absorption, even with low water contents, microwave-assisted extraction (MAE) showed better green performance employing lower plant matter/water ratios (1 : 2 or 1 : 4) and applying shorter extraction times compared with conventional hydrodistillation (HD). Using the single-mode MAE reactor, in the case of *Cymbopogon nardus*, for a plant matter/water ratio of 1 : 4 the energy efficiency was 1.78 g kW^−1^ h^−1^, applying 0.3 kW for 16.7 min. By way of comparison, for the same extraction time using HD, the corresponding efficiency was only 0.50 g kW^−1^ h^−1^. In experiments with citronella using multimode MAE, the best energy efficiency of 2.53 g kW^−1^ h^−1^ was obtained with a plant matter/water ratio of 1 : 2 applying 1.8 kW of power for 30 min. Single and multimode MAE experiments showed optimum conditions with lower water content. Thus, greater amounts of material can be processed in a shorter time, in accordance with the ideals of a green chemistry. The resulting extractions showed an energy efficiency up to 27 times greater compared with conventional HD, applying the same extraction time.

## Abstract 2

Dielectric properties of plants/water mixtures enables the design of single mode cavities for microwave assisted extraction of essentials oils.

## Graphical abstract

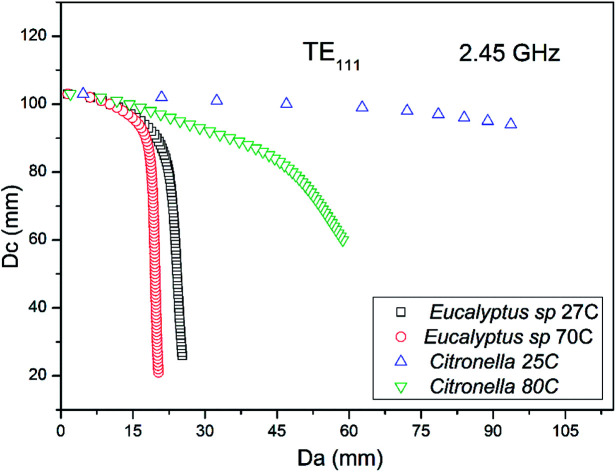

## Introduction

Essential oils or volatile oils, also known as essences, originate from the secondary metabolism of plants and have a complex chemical composition. The terpene, phenolic and alcoholic components of such oils are obtained from the cold-pressing, hydrodistillation or steam distillation of plant material. Essential oils play a remarkable role as organoleptic agents, antioxidants, toxicity reducing agents and food pathogen inhibitors. They are currently the subject of intensive research, as can be seen by the vast literature, with many reviews published recently.^1–5^ These oils exhibit many biological properties and are used in foods, cosmetics and fragrances as well as in the pharmaceutical industry. The following are a few examples: *Clausena dentata* acts as a larvicide^6^ for *Aedes aegypti*; *Myrtus communis* has antioxidant activity;^7^*Lippia gracilis* Schauer demonstrates analgesic, anti-inflammatory and antibacterial activity;^8,9^ oregano is a very effective fungicide^10^ against 12 species of *Aspergillus*; the essential oil of citronella grass (*Cymbopogon nardus* and *Cymbopogon winterianus*) is commonly used as a repellent against insects,^11^ mainly *Aedes* sp., *Coquillettidia perturbans* and *Anopheles quadrimaculatus*; the monkey-pepper plant (*Piper aduncum*) shows efficacy for treating gynecologic diseases along with cytotoxicity and antibacterial activity.^12^ Many species of eucalyptus exhibit antimicrobial^13^ and antioxidant^14^ activity, acting as a larvicide against *Aedes aegypti* and *Aedes albopictus*.

The more traditional techniques used for the extraction of essential oils include hydrodistillation (HD), steam distillation and organic solvent extraction. However, more recently, the application of innovative techniques such as microwave-assisted hydrodistillation (MAHD) and solvent free microwave extraction (SFME) has grown, along with other microwave-assisted techniques, including microwave steam distillation (MWSD) and the microwave-assisted hydrodiffusion and gravity (MWHG) method.^15–19^ The main disadvantages associated with the conventional techniques are the degradation of some volatile compounds (due to long extraction times) and of unsaturated compounds through thermal or hydrolytic effects. Microwave dielectric heating is more effective and selective than conventional heating. It accelerates the energy transfer and provides faster extraction of essential oils (minutes instead of hours), allows the use of lower amounts of solvent, meaning less energy consumption, involves shorter procedures and, most important, leads to a higher purity of the final product.

Microwave-assisted extraction (MAE) has been employed by many research groups who achieved high yields of essential oils (EOs) with high purity and reduced costs.^1–5^ In most of these procedures, MAE processes are carried out in multimode reactors (such as microwave ovens) and only a few authors have employed single-mode MAE reactors.^20^ Akhbari *et al.*,^21^ for example, achieved the optimum conditions for the extraction of essential oil from *Rosemary officinalis* applying 888 W for 85 min, with a yield of 0.7756%. The authors obtained the maximum essential oil quality (55.87%) employing 700 W of microwave irradiation with an extraction time of 68 min (dry plant material without the addition of water). Racoti *et al.*^20^ reported the application of MAHD to fresh ginger root in a multimode reactor and in a TE_10*n*_ single-mode microwave reactor, with 2 kW of variable power. The results obtained in the single-mode cavity showed that higher powers and shorter exposures times did not degrade the oil components. The optimum conditions reported by these authors were 1 kW power and an extraction time of 5 min, affording 0.35 g of oil per 100 g of plant. This performance is high compared with 0.2 g/100 g obtained in 140 min by conventional HD and 0.3 g/100 g in 90 min employing a multi-mode cavity. In the work of Turk *et al.*,^22^ the extraction of the essential oil of *Boswellia* species by MAHD is compared with that conducted by conventional HD. It was observed that microwave (MW) extraction results in a substantial reduction in the extraction rate, that is, 48 min using MAHD (power density of 2 W g^−1^) against 180 min using HD. The energy employed in MAHD was found to be lower by a factor of 2.7 than the energy used in HD. A publication by Gonzalez-Rivera *et al.*^23^ details innovative extraction configurations using a microwave coaxial dipole antenna, where MAHD, SFME and simultaneous ultrasounds and MAHD (US-MAHD) were employed and compared with conventional HD. Their findings in the extraction of essential oil from orange peel showed that with HD the energy consumed per gram of essential oil obtained was 0.83 kW h g^−1^. In comparison, on employing MAHD this value reduces to 0.31 kW h g^−1^ and it is further reduced to 0.28 kW h g^−1^ using the US-MAHD approach, indicating energy savings of more than 60% compared to HD. These findings are in good agreement with those reported in the literature for similar MW assisted approaches.^24,25^ It should be noted that gas chromatography analysis has verified no significant differences in the composition of EOs extracted by MAE methods. Thus, these procedures can be considered as fast, economical, and environmentally friendly extraction methods.

The above few examples demonstrate that the recently introduced innovative processes, mainly employing microwave heating, are very promising, allowing a move towards the application of green chemistry in the extraction of essential oils, as previously suggested by F. Chemat.^26^ These extractions can thus be performed with less energy consumption, reducing the amounts of organic solvents required, shortening the process time, employing abundant raw materials of easy acquisition and, instead of waste, generating by-products with high added value. Since these promising techniques involve the use of microwave dielectric heating, the interaction between microwaves and the materials needs to be better understood, as a starting point to developing better approaches for the green extraction of essential oils. In this regard, few publications in the literature deal with the measurement of the dielectric properties of plants and their mixtures with water as a function of the frequency and temperature.^27,28^ One example is the paper by Racoti *et al.*,^20^ which reports the dielectric constant and loss factor for fresh ground ginger, pressed ginger pulp and ginger juice, measured at room temperature using a dielectric coaxial probe and the cavity perturbation technique at 2.45 GHz.

The determination of the dielectric parameters gives precise information on the interaction of the samples with the electromagnetic radiation and aids the design and operation of single-mode reactors operating at specific frequencies (915 MHz or 2450 MHz). In this context, in this study, the dielectric properties of *Cymbopogon nardus*, *Eucalyptus* sp., *Piper aduncum* and *Piper hispidinervum* were measured using dry plant material and mixtures with water. The dielectric parameters measured were used to solve numerically the transcendental equations that govern single-mode oscillations in cylindrical cavities. A FORTRAN code was developed to solve numerically these equations for the permitted frequencies (915 MHz and 2450 MHz). This allows specific reactors to be designed by calculating the internal radius of the reactor that contains the sample. In the ESI,† the main concepts concerning the dielectric behavior of materials and electromagnetic oscillations in cylindrical cavities are presented. In this study, the MAHD extractions were performed using a single-mode reactor, built to operate in TE_111_ mode, and also using a reactor built to operate in multimode. HD extraction was performed to allow comparison with the MAHD results.

## Materials and methods

### Materials

Water was distilled and deionized (conductivity of 0.05 μS cm^−1^). The *Eucalyptus* sp. leaves were collected from the Federal University of Mato Grosso in Cuiabá (Mato Grosso State, Brazil). The leaves and stalks of *Piper aduncum* and *Piper hispidinervum* were obtained from the Federal University of Acre in Rio Branco (Acre State, Brazil) and *Cymbopogon nardus* (citronella) from the Experimental Farm of EMPAER, located in Acorizal (Mato Grosso State, Brazil).

### Equipment

A water distiller (model 2012, GFL) and a water ultra-purifier (model Direct-Q 3UV, MILLIPORE®) were used in the experiments, along with an infrared humidity analyzer (model IV 2002, GEHAKA®) and a gas chromatograph coupled with mass spectrometer (model QP 5050A, SHIMADZU®). The measurement of the dielectric parameters was performed with an open-ended coaxial probe (HP 85070B, Agilent, Palo Alto, CA, USA) connected to a network analyzer (HP 8753C, Agilent, Palo Alto, CA, USA), in a 101-point frequency sweep from 300 MHz to 13 GHz. The extractions induced by microwave heating were performed in a multimode and single-mode reactors specifically developed for this purpose. The details concerning the microwave reactors are given in the ESI.†

### Methods

The measurement of dielectric properties as a function of the frequency between 0.3 and 13 GHz, in the temperature range of 10 °C to 80 °C, was carried out with samples of dry, crushed and sieved plant material and also with mixtures of this material with ultrapure water in proportions ranging from 1 : 1 to 1 : 8 (plant matter/water). Aliquots of the plant matter were weighed and appropriate amounts of water were added for each plant matter/water ratio. The mixtures were then homogenized prior to the measurements taken at various temperatures. The plant mass in each experiment was sufficient to complete the measuring cell and hence varied according to the individual characteristics of each plant. The specific procedures applied to measure the dielectric parameters were based on the methodology developed in previous studies.^29,30^ The water contents of the plant matter samples were determined by drying under infrared radiation and were 9.8% for *P. aduncum*, 7.9% for *P. hispidinervum*, 6.5% for *Eucalyptus* sp., and 7.5% for citronella.

The extraction of the essential oils by hydrodistillation (HD) employing the Clevenger apparatus and classical heating was performed according to a method described in the literature.^3^ The microwave-assisted extractions were carried out on single-mode and multimode prototypes, varying the plant matter/water ratio, irradiation time, power/mass ratio, type of refrigeration and condensation temperature of the water vapor and essential oil. After completion of the extraction, the essential oils were separated from the water by partitioning with chloroform (3 × 10 mL), dried with anhydrous sodium sulfate, filtered, and stored in amber flasks after evaporation of the chloroform. The chemical composition of the essential oils was determined by gas chromatography coupled to mass spectrometry (GC-MS) in a capillary column of fused silica ATTM-5 (Alltech®, 5% diphenyl and 95% dimethylsiloxane), with 30 m length, 0.25 mm internal diameter and 0.25 μm film, applying conditions described in the literature (with adaptations).^31^ The essential oil components were identified by analyzing the fragments on the mass spectra and by comparing the mass spectra with those in the NIST 11 and 11S libraries available in the apparatus as well as with spectra available in the literature.^31–34^

## Results and discussion

**Fig. 1:**
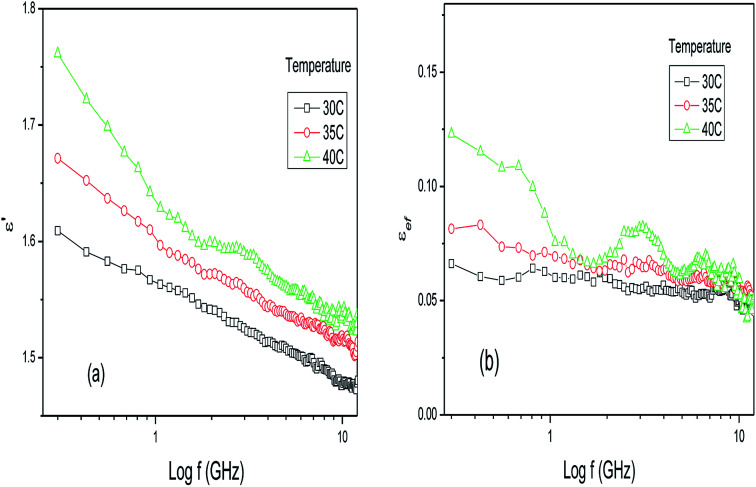
(a) Dielectric constant and (b) loss factor results for dry leaves of *Piper aduncum* as a function of frequency and temperature.

Electrolytic solutions, such as reaction media under base or acid conditions, exhibit complex dielectric behavior that is dependent on the frequency and temperature as well as the concentration of the components of the mixture.^29,30,35^ This behavior originates from the presence of strong electrical fields that are generated due to the presence of ions, affecting the molecular interactions between the solute and the solvent molecules and their respective interactions with applied electrical fields. The same reasoning can be applied to mixtures of plants and water, where many parameters, such as the temperature, density, volume, concentration of water and type of plant, contribute to the global dielectric behavior exhibited by the mixture as a function of the frequency of the applied field. Fig. 1a and b show the dielectric constant and loss factor values for the dry leaves of *Piper aduncum* at three different temperatures in the frequency range of 0.3–13 GHz.

The dielectric constant results in Fig. 1a show small values when compared with ethanol, water or other polar solvents. This is due to the almost non-polar nature of the sample and demonstrates that the dielectric media structurally characterized as complexes, such as biological materials like leaves, are composed of inorganic ions and mixtures of large and small molecules, in this case originating from the primary and secondary metabolic pathways of the plant. Secondary metabolism produces a large number of specific compounds that do not aid in the growth and development of plants but are required for the plant to survive in its environment. Based on their biosynthetic origins, plant secondary metabolites can be divided into three major groups: flavonoids and allied phenolic and polyphenolic compounds, terpenoids and nitrogen-containing alkaloids and sulphur-containing compounds.^36^ In the microwave region, the electrical field component is rapidly reversed and therefore non-polar long-chain molecules are no longer able to easily rotate by a significant amount, before the electrical field is reversed. Furthermore, when the medium is comprised of large polar molecules, parts of these molecules can move relative to the other parts, resulting in one or more distorted polarization processes, each with its own relaxation behavior. The interfaces present in the material provide dipole layers that, when exposed to an electric field, originate interfacial polarization, a phenomenon similar to the Maxwell–Wagner effect, which also exhibits its own relaxation behavior. In Fig. 1a, an increase in the dielectric constant with increasing temperature is observed. As the temperature increases the kinetic energy of the molecules increases and, consequently, they are further apart. This enables a faster response to changes in the electrical field and enhances the realignment process, leading to an increase in the dielectric constant compared to lower temperatures. Fig. 1b emphasizes the complex relaxation behaviors with the presence of many or all of the loss processes in the material and each process contributes to the total magnitude of the dielectric behavior. These contributions lead to a higher loss factor as the temperature increases, as seen in Fig. 1b.

**Fig. 2:**
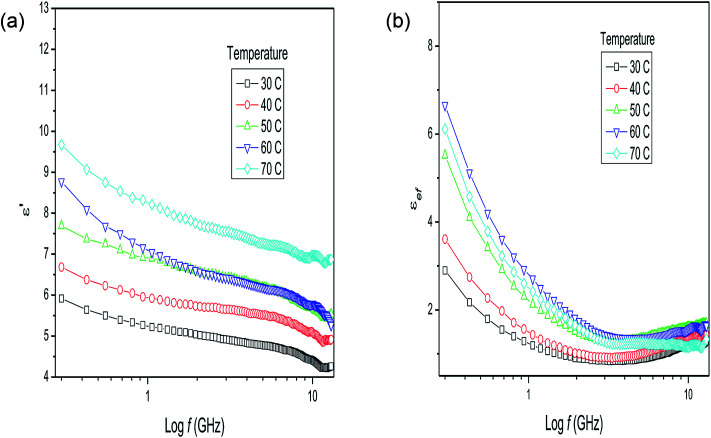
(a) Dielectric constant and (b) loss factor results for *Piper aduncum* (stalks) with the addition of water (1 : 1) as a function of frequency and temperature.

Together with these possible phenomena occurring in materials with a complex structure, the intrinsic effects of the methodology applied to obtain the measurements need to be considered. The homogeneity of the temperature in the material analyzed, for instance, is fundamental to obtaining a reliable measurement. The technical difficulties inherent to the measurement of dry plant matter (*i.e.*, a solid material) are reduced by the addition of water. With the addition of water, the dielectric behavior of the plant matter–water mixture changes markedly, even with a plant matter/water ratio of 1 : 1, as observed in Fig. 2a and b from the dielectric behavior of milled stalks of *P. aduncum* mixed with water. The values for the dielectric constant increase considerably in relation to the dry plant matter, due to the presence of polar water molecules. The mixture of ions, non-polar long chains, and large polar molecules with water leads to an increase in the dielectric constant (Fig. 2a) as the temperature increases, reproducing partially the dielectric behavior of water as a function of the frequency. The fast increase in the loss factor of this mixture, at frequencies below 1 GHz (Fig. 2b), is due to the presence of ions, that is, the values observed for *ε*_ef_ are mainly due to the ionic conduction and the increase in the loss factor at higher temperatures is due to the fact that more and more ions are released from the plant into the water as the temperature increases.

**Fig. 3:**
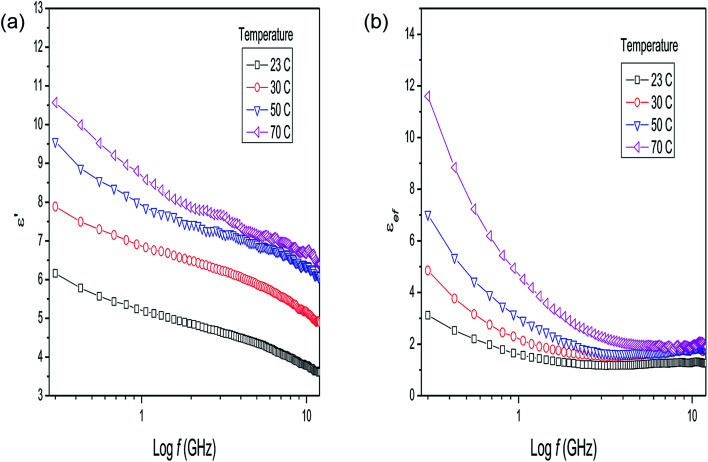
(a) Dielectric constant and (b) loss factor results for *Piper aduncum* (leaves) with addition of water (1 : 1) as a function of frequency and temperature.

**Fig. 4:**
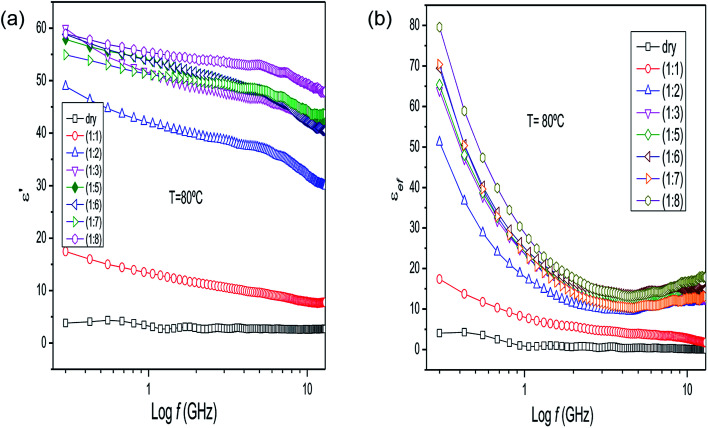
(a) Dielectric constant and (b) loss factor results for *Eucalyptus* sp. (leaves) as a function of frequency and water content at 80 °C.

The dielectric parameters of the mixture of *P. aduncum* leaves with water (1 : 1) as a function of frequency (Fig. 3a and b) show the same trends as the *P. aduncum* stalks with water (Fig. 2a and b), but the loss factor has higher values at low frequencies (<1 GHz). Fig. 3 gives the results for four temperatures, since at certain temperatures some measurements presented large errors and were neglected. The results in Fig. 2b and 3b show an increase in the number of ions in the mixture with temperature, and in the case of the leaves ions are more easily released into the aqueous medium. A clearer understanding of the dielectric behavior of plant matter/water mixtures as the water content increases can be obtained from the measurements for dry leaves of *Eucalyptus* sp. with an increase in the plant matter/water ratio from 1 : 1 to 1 : 8 (Fig. 4a and b). The behavior of the dielectric constant for a plant matter/water ratio above 1 : 2 resembles that of water in the respective frequency range, with values of 50–60 at very low frequencies. In comparison, at 300 MHz, water has a dielectric constant of 63 at 70 °C.

The behavior of the loss factor of these mixtures at 80 °C (Fig. 4b) is more prominent, reaching high values below 1 GHz and increasing with water content, indicating that the ions are more easily released at higher temperatures by the plant matter in the aqueous medium. Therefore, the behavior of *ε*_ef_ is controlled by ionic conduction. These ions are derived from small organic molecules produced from the secondary metabolism of the plant and inorganic ions. The complete set of results for the dielectric properties of the *P. hispidinervum*, *P. aduncum*, *Eucalyptus* sp. and *C. nardus* samples is given in the ESI.†

The dielectric constant, loss factor, loss tangent and penetration depth values at various temperatures for the dry plant matter at two allocated frequencies (0.915 and 2.45 GHz) are shown in Table S1 of the ESI.† The low absorption of electromagnetic energy by the dry plant matter is reflected in the small values for the loss tangent and higher penetration depths. The dielectric properties determine the interaction between the electromagnetic field and the continuous medium, which results in different penetration depths for each plant, as shown in Table S1.†

For all of the dry plant matter samples, at both frequencies, the penetration depths show the same general trend, that is, a decrease with increasing temperature (Table S1†). This behavior is mainly due to changes in the loss factor, since the dielectric constant does not change appreciably. However, it should be noted that for the stalks of *P. aduncum* and *P. hispidinervum*, the changes in the dielectric constant and loss factor led to small variations in the penetration depths of these samples, a specific feature of these plants. Table S2 of the ESI† demonstrates the abrupt changes in the dielectric properties of the samples with the addition of water (plant matter/water ratio of 1 : 1). The penetration depths decrease almost an order of magnitude relative to the dry plant matter, due to the release of different types of ions in the aqueous medium. This increases the loss factor by ionic conduction and as the temperature increases more ions are released, which in turn decreases the penetration depths at higher temperatures. An exception was observed for the stalks of *P. hispidinervum* where, for temperatures above 50 °C, there was an increase in the penetration depths at both frequencies. Increasing the water content in the plant matter/water mixtures changes markedly the dielectric parameters, as demonstrated in Fig. 3a and b. Table S3 of the ESI† shows the temperature-dependence of the dielectric properties for the *P. aduncum* leaves, in a 1 : 8 mixture, at the frequencies of 0.915 and 2.45 GHz. At both frequencies the values for the dielectric constant and loss factor increase markedly relative to those found for the leaves of *P. aduncum* with a plant matter/water ratio of 1 : 1 (Table S2†). However, Table S2† shows that the loss tangents for *Piper aduncum* (1 : 1) are in the ranges of 0.30 < tan *δ* < 0.60 at 0.915 GHz and 0.20 < tan *δ* < 0.33 at 2.45 GHz. With a higher water content (1 : 8), Table S3† shows that at 0.915 GHz the loss tangent increases considerably (mostly due to ionic conduction), but at 2.45 GHz the loss tangent remains almost in the same range (0.20 < tan *δ* < 0.50), since there is a smaller contribution from ionic conduction at higher frequencies. The observed decrease in the penetration depth, at both frequencies (Table S3†), as the temperature increases, reflects the predominance of the relaxation process (due to the large number of molecules present in the 1 : 8 mixture), rather than the ionic contribution originating from the ions released into the aqueous medium by the plants.

A knowledge of the dielectric properties of dry plant matter, or its mixtures with water, obtained from measurements, aids the microwave-assisted extraction of essential oils in specifically-designed microwave reactors operating at an optimum frequency [within those permitted by the Industrial, Scientific, and Medical (ISM) regulations]. Specifically, a knowledge of the changes in the *ε*′ and *ε*_ef_ values as a function of frequency and temperature allows the transcendental equations to be solved (see the ESI†). This allows the construction of cylindrical cavities for TM_010_ and TE_111_ modes at the two allocated ISM frequencies (0.915 and 2.45 GHz), at the desired temperatures.

**Fig. 5:**
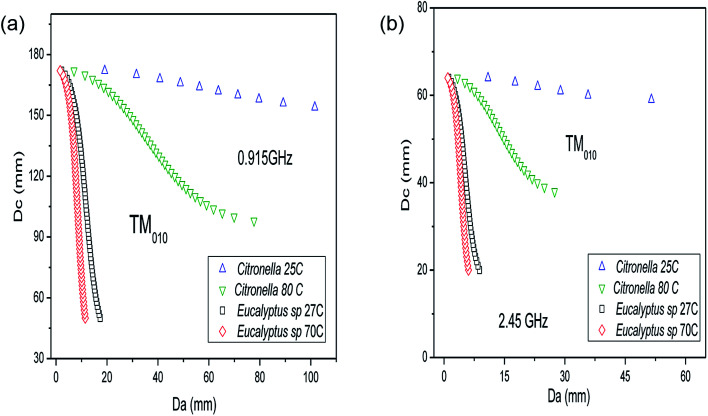
Internal diameter of a TM_010_ cavity (*D*_c_) as a function of the dielectric diameter (*D*_a_) for the mixture of *Cymbopogon nardus* leaves and water (1 : 1) and *Eucalyptus* sp. and water (1 : 8) at two different temperatures for (a) 0.915 GHz and (b) 2.45 GHz.

**Fig. 6:**
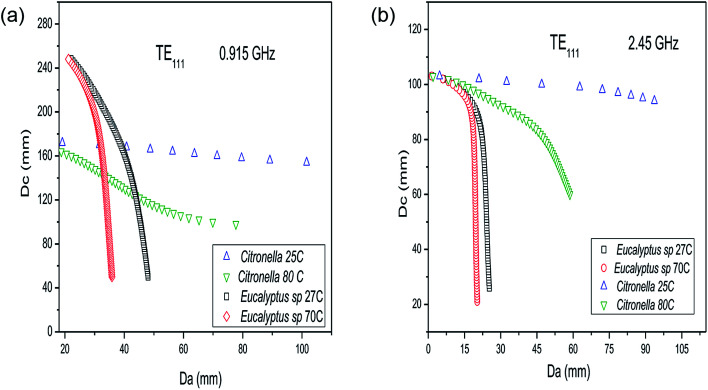
Internal diameter of a TE_111_ cavity (*D*_c_) as a function of the dielectric diameter (*D*_a_) for the mixture of *Cymbopogon nardus* leaves and water (1 : 1) and *Eucalyptus* sp. and water (1 : 8) at two different temperatures for (a) 0.915 GHz and (b) 2.45 GHz.

Fig. 5 shows the results for the solution of eqn (4) (see ESI†) for the TM_010_ resonant mode, affording the internal cavity diameter (*D*_c_) as a function of the dielectric diameter (*D*_a_), employing the dielectric parameters of the mixtures of *C. nardus*/water (1 : 1) and *Eucalyptus* sp./water (1 : 8) at two different temperatures. As seen in Fig. 5, for both frequencies, the dependence of the dielectric properties on the temperature affects strongly the relationship between *D*_c_ and *D*_a_. For the *Eucalyptus* sp., a high water content increases the dielectric constant and loss factor of the mixture, which decreases the allowed dielectric diameters (*D*_a_). For the processing of *C. nardus*/water (1 : 1) at 915 MHz (Fig. 5a) in a cavity with *D*_c_ = 100 mm, for instance, the dielectric diameter (*D*_a_) is 69.8 mm at 80 °C, but for *Eucalyptus* sp./water (1 : 8) at 70 °C, the correct *D*_a_ is 8.7 mm. The crucial information obtained from the results in Fig. 5 is the fact that a lower water content is associated with a larger dielectric diameter and thus the amount of material that can be processed is greater, which is in accordance with the ideals of green chemistry. As can be seen in Fig. 5a and b, the result for the allowed dielectric diameter (*D*_a_) for mixtures with high water content is less sensitive to temperature changes. Also, the amount of material that can be processed in cylindrical reactors is greater when using the lower frequency (0.915 GHz). Fig. 6 shows the changes in the relationship between *D*_c_ and *D*_a_ when considering the cylindrical reactor operating in the TE_111_ mode.

For a cylindrical reactor operating in TE_111_ mode, the results for the allowed dielectric diameter is also less sensitive to temperature changes for the mixture with a higher water content, that is, *Eucalyptus* sp./water at 1 : 8. For TE_111_, at 2.45 GHz a cavity diameter (*D*_c_) of 98.0 mm provides a dielectric diameter of *D*_a_ = 12.9 mm for *Eucalyptus* sp./water (1 : 8) at 70 °C, whereas for *C. nardus*/water (1 : 1) at 25 °C the allowed diameter is *D*_a_ = 72.0 mm, which reduces to *D*_a_ = 18.7 mm at 80 °C. However, as seen in Fig. 6b, with a cavity diameter of 60 mm it is possible to process *C. nardus*/water (1 : 1) at 80 °C using a dielectric diameter of 58.7 mm, resulting in a cavity almost filled with the sample. Therefore, employing the TE_111_ mode, it is also possible to process almost the same amount of material as in the TM_010_ using mixtures with lower water content. The solution of eqn (4) and (5) given in the ESI† was carried out for representative samples of water mixtures with *P. hispidinervum*, *P. aduncum*, *C. nardus* and *Eucalyptus* sp., with different quantities of water and at different temperatures. These data are provided in the ESI.† For all samples, the solutions found demonstrate that larger dielectric diameters are ideal only for mixtures with low water content. Also, as indicated in Fig. 5 and 6, the dielectric diameters are higher at low temperatures, decreasing considerably at high temperatures. This limits the amount of material that can be processed, and thus single-mode reactors are suitable for processing filamentary materials in small amounts, that is, in lab scale. Therefore, solving the resonant conditions allows the design and construction of specific reactors for processing a given material at the desired temperature, optimizing the overall process. Further details regarding the results for the internal diameters of the cavities are given in the ESI.† For practical purposes, in this study, two cylindrical reactors were built, one multimode and one TE_111_ single mode, both operating at 2.45 GHz. From the experimental point of view, for the MAE processes it is not feasible to build reactors with small internal diameters (*D*_a_ < 20 mm) that are in accordance with the results shown in Fig. 5 and 6 and with the small penetrations depths found for the plant matter/water mixtures reported in Tables S2 and S3.† Hence, the dimensions of the single-mode reactor built are *D*_c_ = 99.5 mm and *D*_a_ = 70 mm and it is expected that the reactor will not operate under optimum conditions as a single-mode cavity. Furthermore, the small penetration depths of the mixtures shown in Tables S2 and S3† indicate that the total oil yield is greatly dependent on the distribution of the plant material and hence the experiments were performed with stirring of the samples in order to achieve uniformity in the temperature distribution and avoid the appearance of hot spots.

The microwave-assisted extraction of essential oils of *C. nardus* was performed in the single-mode reactor (see ESI†) and the results for a set of 22 experiments are given in Table 1, together with the results using conventional HD with the Clevenger apparatus and conventional heating (experiment 23). In experiment 23, the essential oils were collected after three different extraction times, allowing a comparison with the MAE extraction results. Also, in experiment 23, the oil mass values represent the mass collected at each time added to that collected previously. The same procedure was adopted for the results of the HD experiments reported in Tables 2 and 3.

**Table 1:** Microwave-assisted extraction of essential oil of *Cymbopogon nardus* performed in the single-mode reactor

Experiment	Plant mass (g)	Essential oil (g)	Yield (%)	Plant/water ratio	Power (W)	Time (min)	Energy efficiency g kW^−1^ h^−1^
1^a^	20.0858	0.1147	0.571	(1 : 3)	300	16 : 40	1.376
2^a^	20.0361	0.1484	0.741	(1 : 4)	300	16 : 40	1.781
3^a^	20.0702	0.1253	0.624	(1 : 5)	300	16 : 40	1.503
4^a^	20.0015	0.1014	0.507	(1 : 5)	300	13 : 00	1.560
5^a^	20.0113	0.1214	0.607	(1 : 10)	300	16 : 40	1.457
6^a^	20.0077	0.1191	0.595	(1 : 4)	390	15 : 00	1.221
7^a^	20.0028	0.0835	0.417	(1 : 4)	510	08 : 20	1.179
8^a^	20.0029	0.0939	0.469	(1 : 4)	510	11 : 40	0.947
9^b^	20.0098	0.0691	0.345	(1 : 4)	300	11 : 50	1.168
10^b^	20.0517	0.0396	0.197	(1 : 5)	300	10 : 00	0.792
11^b^	20.017	0.0517	0.258	(1 : 6)	300	10 : 00	1.034
12^c^	20.0214	0.0373	0.186	(1 : 4)	300	10 : 00	0.746
13^c^	20.0957	0.0246	0.122	(1 : 5)	300	10 : 00	0.492
14^c^	20.0015	0.0289	0.144	(1 : 6)	300	10 : 00	0.578
15^c^	20.0265	0.0294	0.147	(1 : 5)	390	10 : 00	0.452
16^d^	20.0479	0.0586	0.292	(1 : 6)	390	10 : 00	0.901
17^d^	20.0208	0.135	0.674	(1 : 6)	510	10 : 00	1.588
18^d^	20.0283	0.1446	0.722	(1 : 6)	600	10 : 00	1.446
19^e^	20.0431	0.1336	0.666	(1 : 6)	600	10 : 00	1.336
20^e^	20.0624	0.1309	0.652	(1 : 7)	600	10 : 00	1.309
21^e^	20.0453	0.1592	0.794	(1 : 7)	600	12 : 09	1.310
22^e^	20.0334	0.1381	0.689	(1 : 7)	600	11 : 00	1.255
23^f^	20.00	0.0114	0.057	(1 : 10)	82	16 : 40	0.500
		0.0296	0.148		82	30 : 00	0.722
		0.169	0.845		82	180	0.687

a A Clevenger apparatus was adapted to the reactor.

b A trap dipped in liquid nitrogen was adapted to the reactor.

c The reactor was connected to a condenser using a thermostatic bath with a temperature of 0 °C.

d Same as “*c*” and ice bath in the collection vial.

e The reactor was connected to a straight condenser using a chiller condensation system with a temperature of −10 °C.

f Hydrodistillation experiment with Clevenger apparatus and conventional heating.

**Table 2:** Microwave-assisted extraction of essential oil of *Cymbopogon nardus* performed in the multimode reactor

Experiment	Plant mass (g)	Essential oil (g)	Yield (%)	Plant/water ratio	Power (W)	Time (min)	Energy efficiency g kW^−1^ h^−1^
1	200.75	0.7417	0.369	1 : 1	900	120	0.412
2	200.02	1.3686	0.684	1 : 5	900	120	0.760
3	200.15	1.3197	0.659	1 : 10	900	120	0.733
4	200.29	2.8502	1.423	1 : 1	1800	60	1.584
5	200.09	2.2733	1.136	1 : 2	1800	30	2.526
6	200.18	2.0407	1.019	1 : 3	1800	30	2.267
7	200.06	2.0415	1.020	1 : 4	1800	30	2.268
8	200.04	1.981	0.990	1 : 5	1800	30	2.201
9	200.01	1.8986	0.949	1 : 6	1800	30	2.109
10	200.21	1.8386	0.918	1 : 7	1800	30	2.043
11	200.16	1.8497	0.924	1 : 8	1800	30	2.055
12	200.08	1.9361	0.968	1 : 9	1800	30	2.151
13	200.1	1.9272	0.963	1 : 10	1800	30	2.141
14	200.18	1.8537	0.926	1 : 1	2460	30	1.508
15	200.1	2.3543	1.177	1 : 2	2460	30	1.914
16	200.03	2.5473	1.273	1 : 3	2460	30	2.071
17	200.03	2.3782	1.189	1 : 4	2460	30	1.933
18	200.08	2.2126	1.106	1 : 5	2460	30	1.798
19	200.03	2.2421	1.121	1 : 6	2460	30	1.823
20	200.06	2.3322	1.166	1 : 7	2460	30	1.896
21	200.2	2.4473	1.222	1 : 8	2460	30	1.989
22	200.16	2.2285	1.113	1 : 9	2460	30	1.811
23	200.13	2.0545	1.027	1 : 10	2460	30	1.670
24^a^	200.01	0.0245	0.012	(1 : 10)	528	30	0.093
		0.6108	0.305		421	60	1.451
		1.1545	0.577		351	120	1.644
		1.318	0.659		298	180	1.474

a Hydrodistillation experiment with Clevenger apparatus and conventional heating.

**Table 3:** Microwave-assisted extraction of essential oil of *Eucalyptus* sp. performed in the multimode reactor

Experiment	Plant mass (g)	Essential oil (g)	Yield (%)	Plant/water ratio	Power (W)	Time (min)	Energy efficiency g kW^−1^ h^−1^
1	200.12	0.2578	0.129	1 : 1	2700	5	1.146
2	200.11	0.9959	0.498	1 : 1	2700	10	2.213
3	200.01	0.3489	0.174	1 : 1	3600	5	1.163
4	200.00	0.4975	0.249	1 : 1	3600	10	0.829
5	200.14	0.8665	0.433	1 : 1	3600	15	0.963
6	200.17	0.5162	0.258	1 : 1	4500	5	1.376
7	200.00	0.8776	0.439	1 : 1	4500	10	1.170
8	200.04	0.2816	0.141	1 : 2	5400	5	0.626
9	200.16	0.6273	0.313	1 : 2	5400	10	0.697
10	200.07	0.8984	0.449	1 : 2	5400	15	0.665
11^a^	200.25	0.0020	0.001	(1 : 10)	478	30	0.008
		0.3406	0.170		390	60	0.873
		0.7649	0.382		344	120	1.112
		1.0357	0.517		327	180	1.055

a Hydrodistillation experiment with Clevenger apparatus and conventional heating.

The experiments with microwaves were performed using adaptations to the single-mode reactor based on three conceptions: the first with a Clevenger, the second with a straight condenser and the third with a trap dipped in a Dewar flask filled with liquid nitrogen, as indicated in Fig. S2 of the ESI (single mode F_A_, F_B_, F_C_, as indicated in Fig. S2†). During the extraction time employing the system single mode F_A_ (Fig. S2†), the condensed water was separated from the essential oil and then returned to the reactor. When employing the system single mode F_B_ (Fig. S2†), the extraction took place without the return of water to the reactor, that is, the water was drained constantly from the reactor. As indicated in Table 1, the energy efficiency of the MAE process is influenced by several variables, including the extraction time, plant matter/water ratio and microwave power, as well as the temperature of the condensation system for the water vapor and essential oils. The best results, according to the principles of green chemistry, are those where the highest yields per kW per h are obtained, in other words, the lowest energy consumption per gram of essential oil, and the lowest water content is employed. Accordingly, better results were obtained in experiments 1 to 5, using the Clevenger-type design adapted to the reactor, compared to the other experiments using trapping or a straight condenser, demonstrating that the return of the condensed water to the reactor is an important factor in the process. Technical difficulties, mainly related to the stirring of the samples, prevented experiments with plant matter/water ratios of 1 : 1 and 1 : 2 to be performed. The best result was obtained in experiment 2, with an energy efficiency of 1.781 g kW^−1^ h^−1^, that is, a consumption of 0.561 kW per h per gram, representing a reduction by almost a factor of 4 compared to the conventional extraction method (experiment 23 in Table 1 has a consumption of 2.00 kW per h per gram) in the same extraction time. The above results are in agreement with those reported by Racoti *et al.*^20^ for fresh ginger root using MAHD performed by TE_10*n*_ in a single-mode microwave reactor with 2 kW of variable power. The authors obtained 0.35 g of essential oil (EO) from whole shredded ginger in 5 min using 1.0 kW of power, indicating an energy efficiency of 4.20 g kW^−1^ h^−1^. In the same study, the extraction from juice + pressed ginger afforded 0.42 g of EO applying 2.0 kW of power in 2.5 min, thus achieving a high energy efficiency of 5.04 g kW^−1^ h^−1^. However, increasing the microwave power does not necessarily improve the energy efficiency, as seen in experiments 6 to 8 of Table 1. Trapping and condenser experiments (experiments 9 to 22), where the water withdrawn in the extraction does not return to the reactor, provided the best results (experiments 17 to 22) when employing the condenser, where the exit of the water vapor and essential oils from the reactor were enhanced through the use of glassware with larger diameters, that generated a lower flow in the process and, therefore, lower losses of essential oils in the condensation employing a lower temperature. The experiments 12 to 15 exhibited the poorest results, even when compared with the conventional extraction (mainly experiments 13 to 15), due to the higher temperature of the condensation system, which leads to an increase in the oil losses. It can be observed in Table 1 that to improve the results at higher plant matter/water ratios (experiments 17 to 22) an increase in the microwave power is required and shorter extraction times need to be applied. These results suggest that the extraction of essential oils in a microwave reactor can also be performed without the return of water to the reactor. However, from the green chemistry point of view it becomes a less attractive process because, although it occurs in a shorter time, a greater amount of water is used, generating a greater amount of residue. Although the single-mode reactor is not optimized to process the *C. nardus* samples at high temperatures, in accordance with the results shown in Fig. 6b, Table 1 demonstrates that, even when working under non-ideal conditions, the MAE results are superior to those obtained with conventional extraction.

The MAE experiments were also performed in the multimode reactor (Fig. S2†), where the plant masses were 10 times higher than those used in the single-mode reactor. Table 2 shows the results for a set of MAE experiments and for conventional extraction (HD; experiment 24), where the oils were collected at four different times, allowing a comparison with MAE extraction at the same extraction time.

The first set of MAE experiments (1 to 3) employing 900 W for 2 h gave very poor results, independently of the plant matter/water ratio, with very low energy efficiency (<0.76 g kW^−1^ h^−1^) compared with conventional extraction with the same extraction time (1.644 g kW^−1^ h^−1^), indicating that high microwave power should not be applied for long extraction times. This finding becomes evident in experiment 4, where the power doubled but the time of extraction was halved, resulting in more than twice the energy efficiency compared with experiments 1 to 3. Experiment 4 also presents a better result for the energy efficiency (1.584 g kW^−1^ h^−1^) compared with that obtained with conventional extraction applying the same extraction time (1.451 g kW^−1^ h^−1^). In experiments 5 to 13 the extraction time was reduced to 30 min and the best result was obtained in experiment 5 with a plant matter/water ratio of 1 : 2. In this case the energy efficiency (2.526 g kW^−1^ h^−1^) is 27 times greater compared with conventional extraction applying the same extraction time (0.093 g kW^−1^ h^−1^). Conventional extraction is inefficient in the first 30 min since most of the applied energy is used to heat the mixture to reach the boiling point. Thus, a longer time is required to transfer the heat from the source to the mixture. On the other hand, with the use of microwaves, volumetric heating occurs with an almost vertical temperature ramp. According to the MAE results for experiment 5, the amount of *C. nardus* oil produced in 30 min was 2.2733 g, with a consumption of 0.45 kW h, while applying the HD method (experiment 24) 1.318 g of oil was obtained in 3 h consuming 0.894 kW h. These results indicate energy savings of around 50% in a shorter process using less water and producing a lower amount of waste in the case of MAE. The results for experiments 5 to 13 demonstrate that MAE extraction should be performed with the lowest water content possible, in agreement with the green chemistry concept. The best results in Table 2 are comparable with those obtained by Gonzalez-Rivera *et al.*^23^ using a microwave coaxial dipole antenna. Applying MAHD to the extraction of essential oil from orange peel they achieved an energy efficiency of 3.225 g kW^−1^ h^−1^ and using US-MAHD this increased to 3.571 g kW^−1^ h^−1^, that is, the energy consumption values were 0.310 kW h g^−1^ and 0.280 kW h g^−1^, respectively. In comparison, the best result in Table 2 (experiment 5) corresponds to an energy consumption of 0.396 kW h g^−1^. However, it should be noted that these results relate to different samples (citronella and orange peel) and the concentrations of EOs are not the same and, more importantly, the dielectric properties differ. Thus, the comparison is only approximate, highlighting the general trend in the efficiency of the MAE process compared to conventional extraction, when applied to different samples. In experiments 14 to 23 the microwave power was increased to 2460 W applying the same extraction time, confirming the small change in the energy efficiency values as the water content in the plant matter/water mixture increases. The best value for the energy efficiency (2.071 g kW^−1^ h^−1^) was obtained in experiment 16, employing a plant matter/water ratio of 1 : 3. Here the efficiency was 22 times greater than the value obtained with conventional extraction with the same extraction time. Even considering the lowest result obtained in this set (experiment 23), using a plant matter/water ratio of 1 : 10, the energy efficiency was 18 times greater than that obtained in the conventional extraction.

In addition, a third set of MAE experiments was carried out to extract the essential oil of *Eucalyptus* sp., employing plant/water ratios of 1 : 1 and 1 : 2 and high MW powers with shorter extraction times, using the multimode reactor (Fig. S2†). Table 3 shows the results for ten MAE experiments and one HD experiment (no. 11), employing conventional heating with the essential oil collected at three different times.

As evidenced in Table 2, for the HD extraction of *C. nardus* oil, in the first 30 min, the complete inefficiency of HD is clear in Table 3, since 0.239 kW h is required to produce 0.0019 g of oil, while in experiment 1, on applying MAE for 5 min, 0.2578 g of oil is obtained with 0.225 kW h. The best result for the energy efficiency (2.213 g kW^−1^ h^−1^ in 10 min) was obtained in experiment 2, this being more than twice the value obtained applying conventional extraction for 3 h. The energy consumption of experiment 2, for a 10 min extraction, was 0.45 kW h, while on applying HD for 3 h it was 0.981 kW h, showing an energy saving of 46%. This verifies that MAE, besides being a very fast process, requires smaller amounts of water and thus generates less waste. It can be observed in Table 3, as a general trend, that the energy efficiency is higher for shorter extraction times, as the MW power increases. However, with the use of very high power (5400 W) the energy efficiency drops to very low values. In fact, experiments 8–10, performed with short extraction times (5 to 15 min), presented poor energy efficiency (around 0.66 g kW^−1^ h^−1^), with values very similar to those obtained with HD in 1 h. On comparing the MAE (experiment 10) with HD, it can be observed that MAE consumes 1.35 kW h to produce 0.898 g of EO in 15 min while HD consumes 0.981 kW h to produce 1.040 g of EO in 3 h. Thus, even considering the poorest results obtained, the advantages of the MAE process, that is using a lower amount of water and generating less waste, are evident. In summary, besides the MAE process being more economical and environmentally friendly (less solvent, less waste), an important factor is the possibility that the proposed innovative cylindrical multimode reactors can be easily built in industrial scale. Finally, the gas chromatography analysis performed on all of the essential oil samples obtained with the MAE process, showed no appreciable change in the composition of the EOs extracted. In addition, the coloration and odor of the essential oil samples were identical to those obtained by conventional heating. This suggests that thermal degradation of the plant matter or of the essential oils did not occur. The results for the chromatographic analysis and the chemical composition of the EOs extracted are presented in the ESI.†

## Conclusions

A knowledge of the dielectric properties of dry plant matter and its mixtures with water as a function of frequency and temperature allows specific resonant cavities to be built for the extraction of oil *via* the MAE process. The plant matter/water mixtures consist of ions of low polarity, non-polar long chain hydrocarbons, and large polar molecules that exhibit an increasing dielectric constant as the temperature increases, reproducing partially the dielectric behavior of water as a function of frequency. The penetration depths of the plant matter/water mixtures decrease by almost an order of magnitude relative to the dry plant matter. This is due to the release of different types of ions in the aqueous medium, leading to a high absorption of MW and, consequently, a low water content must be used in the MAE process. The results obtained with the innovative reactors showed the influence of the plant matter/water ratio, extraction time, microwave power and the temperature of the condensation system for the water vapor and essential oils on the MAE process. This approach is in accordance with the principles of green chemistry, providing higher yields per kW per h of power employed and thus lower energy consumption per gram of essential oil obtained, and allowing a lower sample water content with minimal generation of waste. Furthermore, MAE equipment with the proposed innovative cylindrical multimode reactors, involving safe operability and faster processing, are easily constructed in industrial scale.

## Conflicts of interest

The authors declare no competing financial interest.

## Supplementary Material

RA-009-C8RA08727J-s001
