# Supplementary material for: Measuring dielectric properties for microwave-assisted extraction of essential oils using single-mode and multimode reactors
Source: RSC Adv. 2019 Feb 12;9(10):5259–69. doi: 10.1039/c8ra08727j (PMC9060669; doi:10.1039/c8ra08727j)
Supplement: RA-009-C8RA08727J-s001 [file RA-009-C8RA08727J-s001.pdf]

Electronic Supplementary Material (ESI) for RSC Advances.
This journal is © The Royal Society of Chemistry 2019

Measuring Dielectric Properties for Microwave-Assisted Extraction of Essentials Oils via single mode and multimode reactors

Carlos A. Parizotto,[a] Evandro L. Dall'Oglio,[a*] Leonardo G. de Vasconcelos[a], Paulo T. de Sousa Jr.,[a] Eduardo G. R. Taques Filho[a] , Carlos Alberto Kuhnen[b]

[a] Departamento de Química Universidade Federal do Mato Grosso Av. Fernando Corrêa da Costa s/n, Coxipó,Cuiabá – MT, CEP:78090-600; [b]Departamento de Física – Universidade Federal do Santa Catarina; Campus Universitário Trindade Florianópolis-SC - CEP 88040-970

SUPLEMENTARY DATA

I. Dielectric Properties

Cylindrical single-mode reactors via measured dielectric properties ………..S2 – S5

References ……………………………………………………………….S6

Equipments ………………………………………………………………. S6 -S7

Measurements

Pure water ………………………………………………………………**S8**

Dry Plants ………………………………………………………………**S9 – S12**

Plant/water mixtures ……………………………………………………**S13- S16**

Tables of dielectric properties …………………………………………**S17-S19**

II. Diameters of the dielectric samples in cylindrical cavities ……………… S20 -S32

Dielectric properties of pure liquids, ethanol, methanol and glycerin ……………**S20 -S21**

Diameters of the dielectric samples: ethanol, methanol and glycerin ………….. **S22 – S27**

Diameters of the dielectric samples: plant/water mixtures ……………………… **S28 – S32**

III. Chromatograms of the Essentials oils extracted by MAE ……………… S33 -S37

Chromatograms of the experiments of table 4 …………………………………. **S33**

Chromatograms of the experiments of table 5 …………………………………. **S34- S36**

Chromatograms of the experiments of table 6 …………………………………. **S37**

IV. Chemical Compositions of the Essentials oils extracted by MAE…………S38

Cylindrical single-mode reactors via measured dielectric properties

Nowadays microwave dielectric heating can be viewed as a tool for sustainable green chemistry, being a very promising procedure, which has been used successfully in several branches of chemistry.[1] Microwave energy consists of electromagnetic radiation in the frequency range 0.3 to 300 GHz whose electrical field interacts with ions and molecular dipole moments generating heating by friction. Conventional heating of thick materials is a slow process relying on heat conduction from the outer layers towards the interior. On the contrary, high frequency electromagnetic waves are efficiently transferred to the material and MW energy is absorbed throughout the volume of the sample. When plant is heated via microwaves, the water inside the cells reaches the boiling point, generating steam by raising the gas pressure and subsequent generation of high pressure in the cell wall of the oil gland. The generated internal pressure reaches values such that the wall of the oil gland cell breaks down. This process greatly facilitates the release or leaching of the essential oil from the plant material into the surrounding solvent (water). If the plant material is soaked in polar solvents as water, the process can be greatly optimized. The high temperature of the cell wall of the plant can improve the dehydration process of the cellulose and decrease the resistance of the cell wall to the solvent inlet, which can thus easily penetrate the cell. In most cases, the plant material is soaked into single solvent or a mixture of solvents having high dielectric loss in order to strongly absorb the MW energy. Only in some special cases the plant material must be soaked into solvents which are transparent to microwave, mainly when the crude extract contains highly thermolabile components. These facts undoubtedly demonstrate the importance of the knowledge of the dielectric properties of dry plants and their water mixtures.

Dielectric properties of materials are defined by their relative complex permittivity $\hat{\varepsilon} = \varepsilon' - j\varepsilon_{ef}$ where the real part ε' is the relative dielectric constant and $\varepsilon_{ef} = (\varepsilon'' + \sigma / \omega\varepsilon_0)$ is the dielectric loss factor of the material with σ being the conductivity and ε'' the imaginary part of the relative permittivity that accounts for the dielectric relaxation process.[1-5] In continuous media, electromagnetic energy dissipation occurs mainly by the mechanisms of ionic conduction and dipolar rotation. Inter- and intra-molecular frictions are created by the interaction of ions and dipoles with the oscillating electrical field generating heat throughout the volume of the material. An important parameter in describing the dielectric response of

materials to the applied MW is the loss tangent, which is defined by $\tan\delta = \varepsilon_{ef} / \varepsilon'$. The penetration depth (D_p), the distance at which the amplitude of the electrical field is damped to 1/e = 0.369 of its initial value at the surface of the material is related to the loss tangent by[3,4] $D_p = \frac{c}{\omega}\sqrt{\frac{2}{\varepsilon'}}\left[\sqrt{1+\tan^2\delta}-1\right]^{-1/2}$ and therefore is frequency and temperature-dependent. Therefore, the region of the sample where the electrical field effectively penetrates is denoted by D_p. It defines the volume of the sample where the dielectric heating effectively occurs. Clearly the absorption of MW energy is non-uniform and the non-uniform temperature distribution inside a dielectric material is determined by the penetration depth. In continuous media the strength of the local electrical field depends on the dielectric properties of the medium (ε', ε_{ef}, $tg\delta$), which, in turn, is function of the field frequency and temperature. To evaluate approximately the electrical field, it is necessary to consider the average electromagnetic power, $\bar{P}$ (W), dissipated by the medium. By employing Maxwell´s equations, the power can be obtained as,[3,4]

$$\bar{P} = \frac{1}{2}\omega\varepsilon_0\varepsilon_{ef}\int_V \left|\vec{\mathcal{E}}\right|^2 dv \cong \omega\varepsilon_0\varepsilon_{ef}\mathcal{E}_{rms}^2 V \quad , \qquad (1)$$

where the electrical field, $\vec{\mathcal{E}}$, inside the volume V of the material is substituted by its root mean square (*rms)* value, $\mathcal{E}_{rms}$. In equation (1) ε_0 is the vacuum permittivity (=8.85x10^{-12} F/m), ε_{ef} is the dielectric loss factor and $\mathcal{E}_{rms}$ is the *rms* value of the applied oscillating electrical field (V/m) with frequency f in hertz ($\omega = 2\pi f$, is the angular velocity in rad/s) over the volume V (m^3) of the sample. To calculate the *rms* value of the electrical field it can be considered the average power absorbed by the medium by taking the volume V of the material determined by the penetration depth (D_p), the volume where the medium effectively absorbs the electromagnetic energy, that is $V \cong D_p^3$.

Microwave heating of the materials are done in multimode and single-mode cavities. A disadvantage of multimode cavities is due to the non-uniform distribution of the electrical field, which enhances the non-uniformity distribution of the temperature in the sample. Despite the uniform distribution of the field in single-mode cavities, they have the disadvantage of processing low amount of materials[3] if it is not possible to operate the cavity in a continuous flow mode. In this work, it was considered cylindrical single-mode and

multimode cavities operating as MW reactors for MAE. Since for single-mode cavities the theoretical approach is straightforward, in this work it was considered the Transverse Magnetic (TM) and Transverse Electric (TE) modes in cylindrical cavities with height *h,* where a dielectric sample (radius R_a) is inserted in a hollow cylinder of Teflon (Fig. S1), whose external radius is equal to the internal radius of the cylindrical inox cavity, that is R_t=R_c.

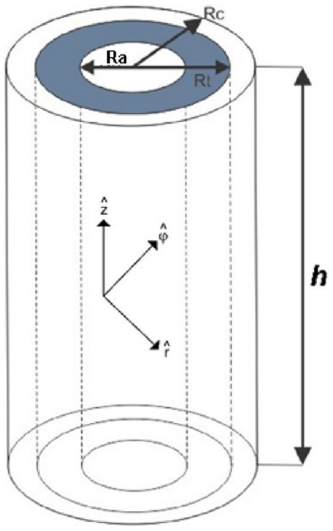

Fig. S1. Cylindrical reactor. R_a is the dielectric sample radius inserted into the reactor. R_t is the external radius of the Teflon. R_c is the internal radius of the inox cavity. $\hat{r}$, $\hat{\varphi}$ and $\hat{z}$ are the unit vectors in cylindrical coordinates.

Since the height *h* of the cylinder can be taken as variable, it will be considered the TM_{010} and TE_{111} fundamental modes. For an empty cavity TM_{010} is the fundamental mode when $h < 2.03R_c$. However, if $h > 2.03R_c$, then the frequency of the TE_{111} mode is lower than that of the TM_{010} mode and, therefore, the TE_{111} mode will be the fundamental mode.[4] When a dielectric is inserted into the region $0 \le r \le R_a$ and the region $R_a \le r \le R_c$ is occupied by Teflon (cavity/load separator) the solutions of the Maxwell equations for the electric and magnetic components of TM_{010} mode are:[4]

$$\begin{aligned} E_{1z} &= E_0 J_0(k_1 r) \\ H_{1\phi} &= -i\sqrt{\frac{\varepsilon}{\mu}} E_0 J_1(k_1 r) \end{aligned} \qquad 0 \le r \le R_a \qquad (2)$$

$$\begin{aligned} E_{2z} &= A_1 J_0(k_2 r) + A_2 Y_0(k_2 r) \\ H_{2\phi} &= -i\sqrt{\frac{\varepsilon_T}{\mu_T}} [A_1 J_1(k_2 r) + A_2 Y_1(k_2 r)] \end{aligned} \qquad R_a \le r \le R_c \qquad (3)$$

where J_0 and J_1 are Bessel functions of zeroth and first order respectively and Y_0 and Y_1 are Neumann functions of zeroth and first order respectively. In the above equations ε and ε_T are

the dielectric constant of the dielectric sample and Teflon respectively ($\varepsilon_T = 2.1$), whereas k_1 and k_2 are given by: $k_1 = \frac{\omega}{c}\sqrt{\varepsilon'}$; $k_2 = \frac{\omega}{c}\sqrt{\varepsilon_T}$. If the sample has an appreciable loss factor, k_1 is given by $k_1 = \frac{\omega}{c}\sqrt{\frac{\varepsilon'}{2}}\left[\sqrt{1+\tan^2\delta}+1\right]^{1/2}$, c is the light velocity and ω is the angular velocity of the microwaves, $\omega = 2\pi f$. The boundary conditions for the electric and magnetic components at $r = R_a$ and at $r = R_c$ (continuity of the tangential components) gives the transcendental equation that controls resonance in the cavity with the presence of the dielectric sample:

$$\sqrt{\frac{\varepsilon'}{\varepsilon_T}}\frac{J_1(k_1R_a)}{J_0(k_1R_a)} = \frac{J_1(k_2R_a)Y_0(k_2R_C) - J_0(k_2R_C)Y_1(k_2R_a)}{J_0(k_2R_a)Y_0(k_2R_C) - J_0(k_2R_C)Y_0(k_2R_a)} \quad (4)$$

Equation 4 tells us that the relationship between cavity radius and dielectric sample radius depends crucially on dielectric properties of the sample (Teflon is a transparent to MW). Therefore, the knowledge the dielectric properties of the sample (by measurement) make it possible, for a given cavity radius, to obtain the allowed value of the sample radius´s by the numerical solution of equation 4. In similar manner, applying the boundary conditions for the electric and magnetic components at $r = R_a$ and at $r = R_c$, on the solutions of the Maxwell equations for the electric and magnetic components of TE_{111} it affords the transcendental equation that governs resonance in such mode:

$$\sqrt{\frac{\varepsilon'}{\varepsilon_T}}\frac{J_1(k_1R_a)}{J_0(k_1R_a) - \frac{J_1(k_1R_a)}{k_1R_a}} = \frac{J_1(k_2R_a)Y_1(k_2R_C) - J_1(k_2R_C)Y_1(k_2R_a)}{[J_0(k_2R_a) - \frac{J_1(k_2R_a)}{k_2R_a}]Y_1(k_2R_C) - J_1(k_2R_C)[Y_0(k_2R_a) - \frac{Y_1(k_2R_a)}{k_2R_a}]} \quad (5)$$

As above, equation 5 shows the strong dependence of sample radius on cavity radius through dielectric properties of the sample inserted in the region $0 \le r \le R_a$. A FORTRAN code was developed to solve numerically equations 4 and 5 for the allowed frequencies 915 MHz and 2450 MHz and using the measured values of the dielectric properties of dry plants and their mixtures. This allows us to build reactors by finding the best internal radius (R_a) for a specific MAHD process, based on the knowledge of dielectric properties of the material to be processed. Based on the knowledge of the dielectric properties of pure liquids, methanol, ethanol and glycerin, preliminary solutions of equations 4 and 5 were found for these liquids, and are given below in this electronic supporting information.

References

1. Leadbeater, N. E. (editor). *Microwave Heating as a Tool for Sustainable Chemistry*, 1st ed.; CRC Press: Boca Raton, FL, 2010.
2. Gabriel, C., Gabriel, S., Grant, E. H., Halstead, B. S. J., Mingos, D. M. P., *Chem. Soc. Rev.,* 1998, **27**(3), 213–223.
3. Metaxas, A. C., Meredith, R. J. *Industrial Microwave Heating*, 2nd ed. London: Peregrinus, 1993.
4. Jackson, J. D. *Classical Electrodynamics*, 2nd ed. John Wiley & Sons Inc., 1972.
5. Gupta, M., Leong, E. W. W., *Microwave and Metals*, John Wiley & Sons (Asia) Pte Ltd, Singapore, 2007.

Equipments

The thermally isolated microwave reactors, were machined in 304 stainless steel and consist of cylindrical resonant cavities (Figure S2). The single mode reactor (F) has an internal diameter of 99.5 mm, in which is inserted a Teflon hollow cylinder (cavity/load separator) (see Fig.S1), with height of 111 mm and inner diameter of 70 mm. The multimode reactor (I), Fig. S2, has the inner diameter of 176 mm and being 175 mm high. The reactors are supplied with electromagnetic radiation by an Alter SM 1150 and SM 1280 microwaves power supplies variable power 0.3−6.0 kW (A in Fig. S2), and Richardson Electronics TM 030 and TM P66 microwave head (B Fig. S2), emitting at 2.45 GHz with the magnetron valve water cooled with a thermostatically controlled bath (DIST DI-980).

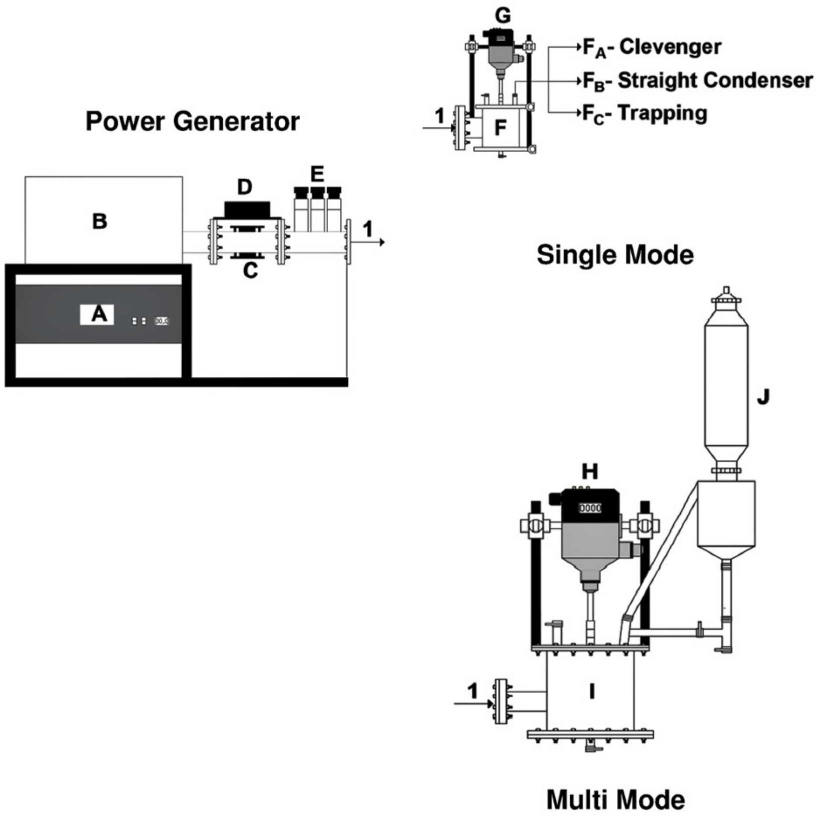

Fig S2: Single and multimode reactors for microwave assisted essentials oils extractions (see text).

The system has a directional coupler/water load (National Electronics D0937) (C Fig. S2), power sensor (Alter RD8400) (D Fig. S2), and waveguide tuner (Alter AG340M3) (E Fig. S2), and the

entire system is controlled by Front Panel 500 software. In the superior flange of single mode reactor, it was adapted a mechanical stirrer with a Teflon paddle and a connector to adapt three different systems to remove water and essential oil in vapor phase. The first is a Clevenger apparatus (F_A Fig. S2), the second is a straight condenser (F_B Fig. S2) and the third is a trapping (F_C Fig. S2). Three different approaches were used to condensate the vapor phase, a thermostatically controlled bath (Tecnal TE 184 model), a Chiller (PolyScience N0772046) and a Dewar flask with liquid nitrogen. In the Clevenger apparatus the condensate water returns to the cavity, but in the other two systems the water is continuously removed from the reactor. Connectors were adapted in both reactors to insert thermal sensors comprised of optic fibers. The temperature in the liquid phase is measured by a Neoptix optic fiber thermometer (model Reflex RFX 378A). In the experiments the energy consumption measurements were performed with a Fluke 434 power quality analyzer. Analysis of the component mixtures was performed with a GC-MS Shimadzu QP 5050A with automatic injector AOC-5000.

1. Pure water

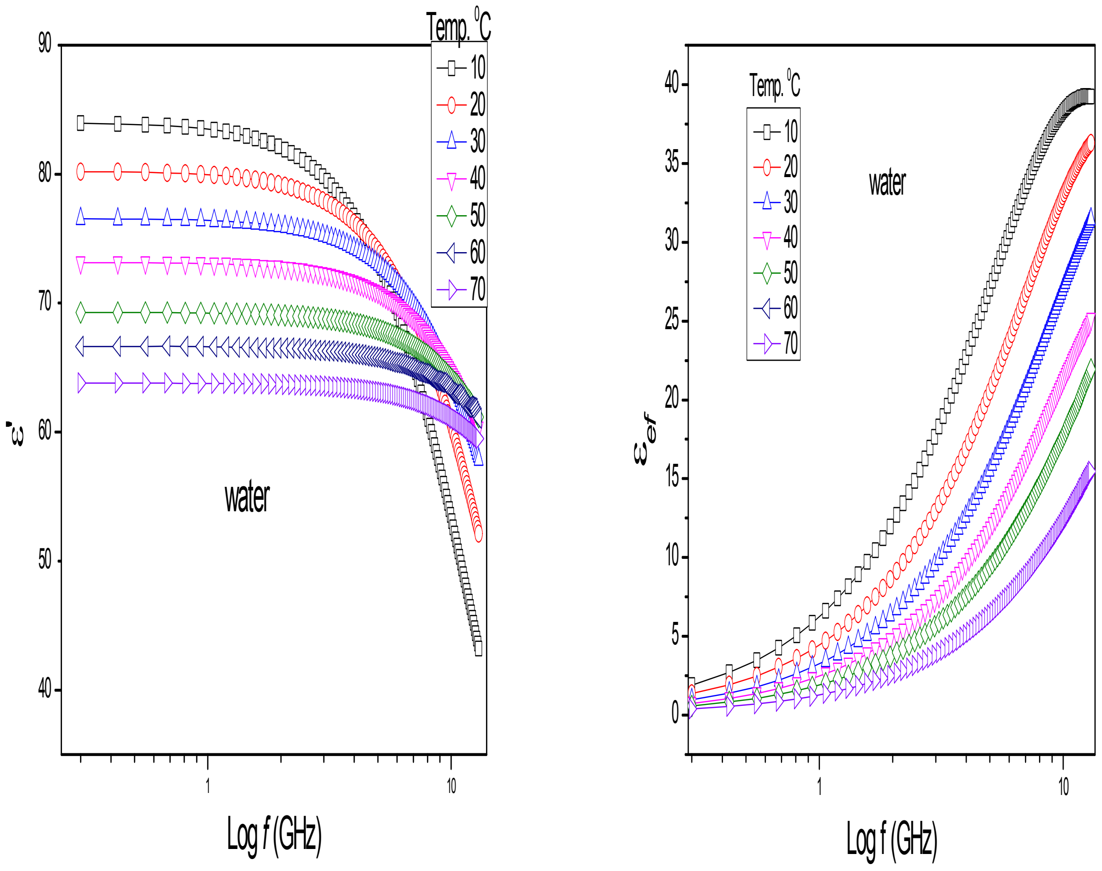

Figure S3. (a) Dielectric constant and (b) loss factor of water as function of frequency and temperature.

2. Dry Plants

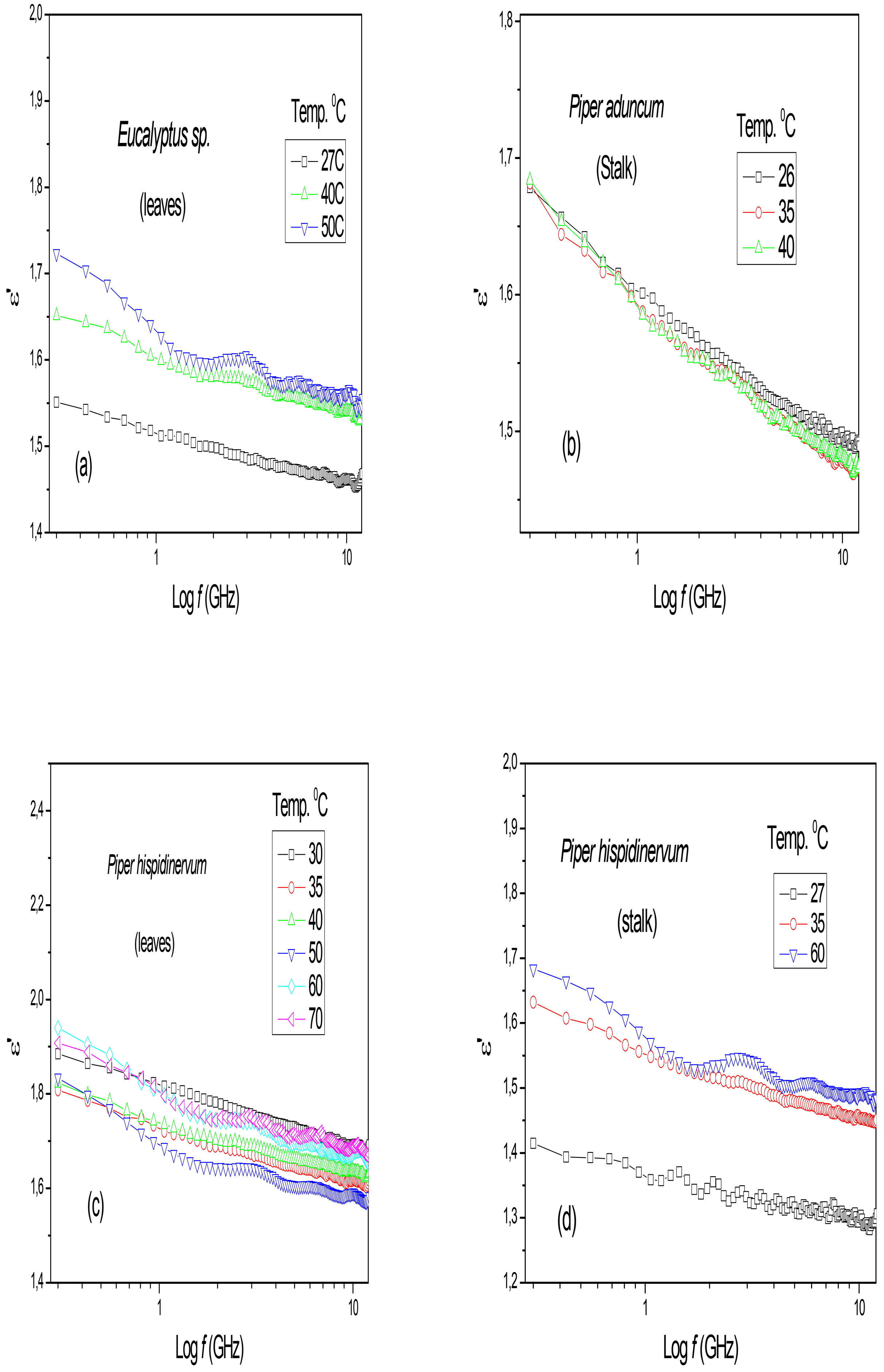
Eucalyptus sp.
(leaves)
Temp. ^{0}C
27C
40C
50C
(a)
ε'
Log f (GHz)
Piper aduncum
(Stalk)
Temp. ^{0}C
26
35
40
(b)
ε'
Log f (GHz)
Piper hispidinervum
(leaves)
Temp. ^{0}C
30
35
40
50
60
70
(c)
ε'
Log f (GHz)
Piper hispidinervum
(stalk)
Temp. ^{0}C
27
35
60
(d)
ε'
Log f (GHz)

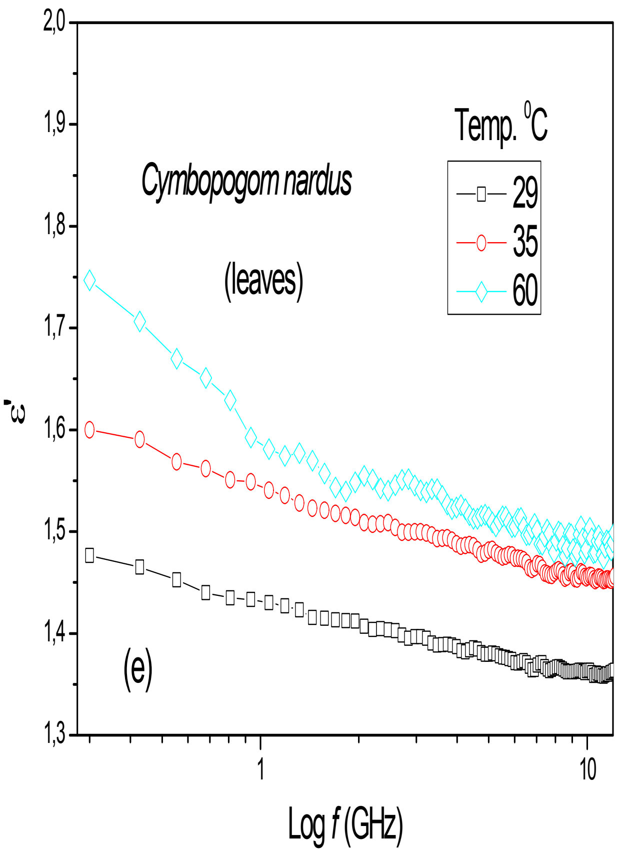

Figure S4. (a) – (e) Dielectric constant of the dry samples as function of frequency and temperature.

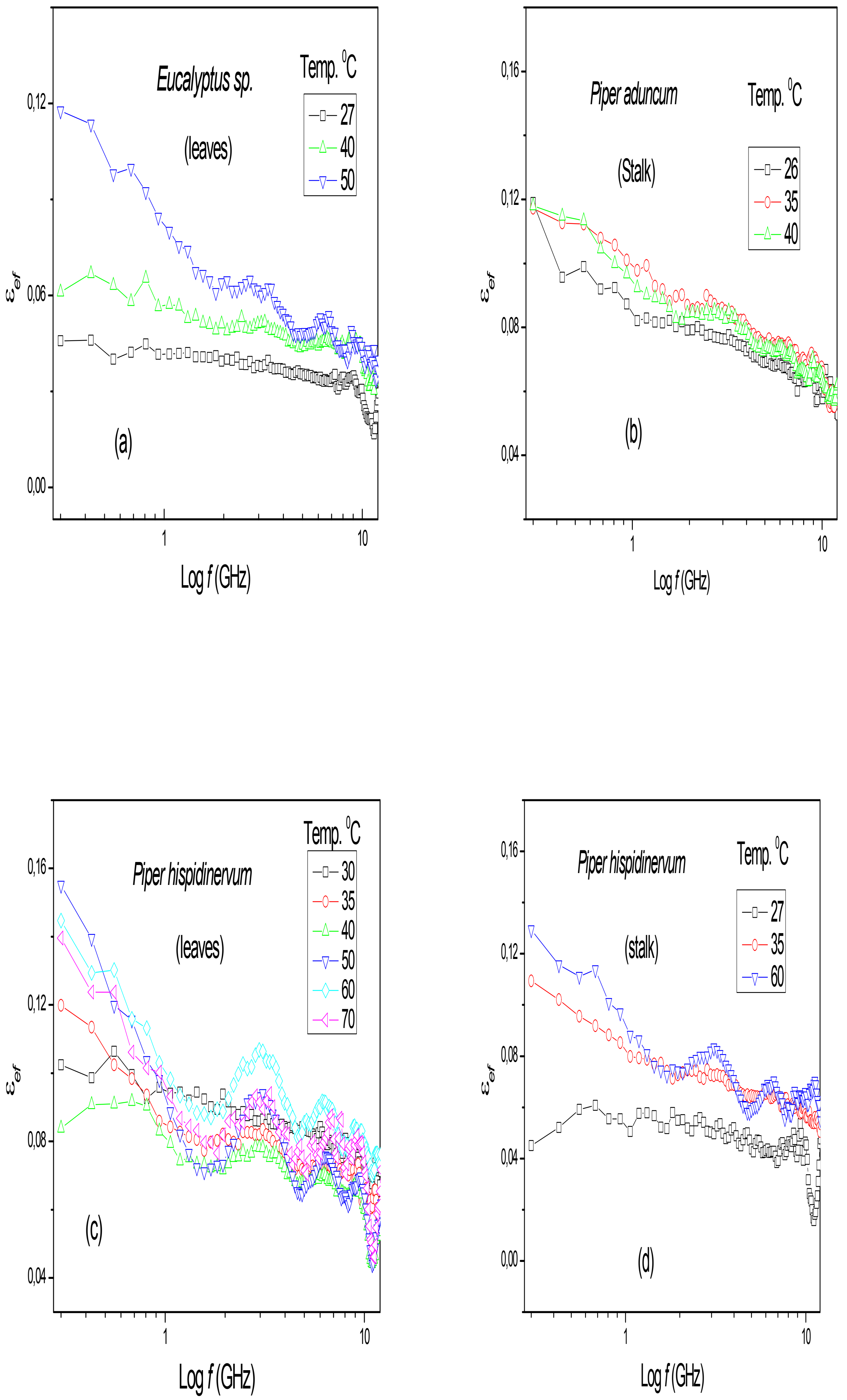
Eucalyptus sp.
(leaves)
Temp. 0C
27
40
50
(a)
Piper aduncum
(Stalk)
Temp. 0C
26
35
40
(b)
Piper hispidinervum
(leaves)
Temp. 0C
30
35
40
50
60
70
(c)
Piper hispidinervum
(stalk)
Temp. 0C
27
35
60
(d)
ε_ef
Log f (GHz)
0,00
0,04
0,06
0,08
0,12
0,16
1
10

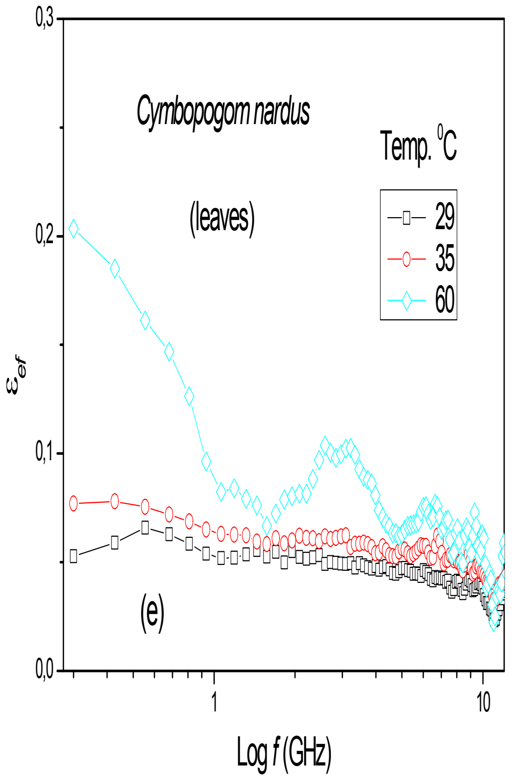

Figure S5. (a) – (e) Dielectric loss factor of the dry samples as function of frequency and temperature.

3. Plants/water mixtures

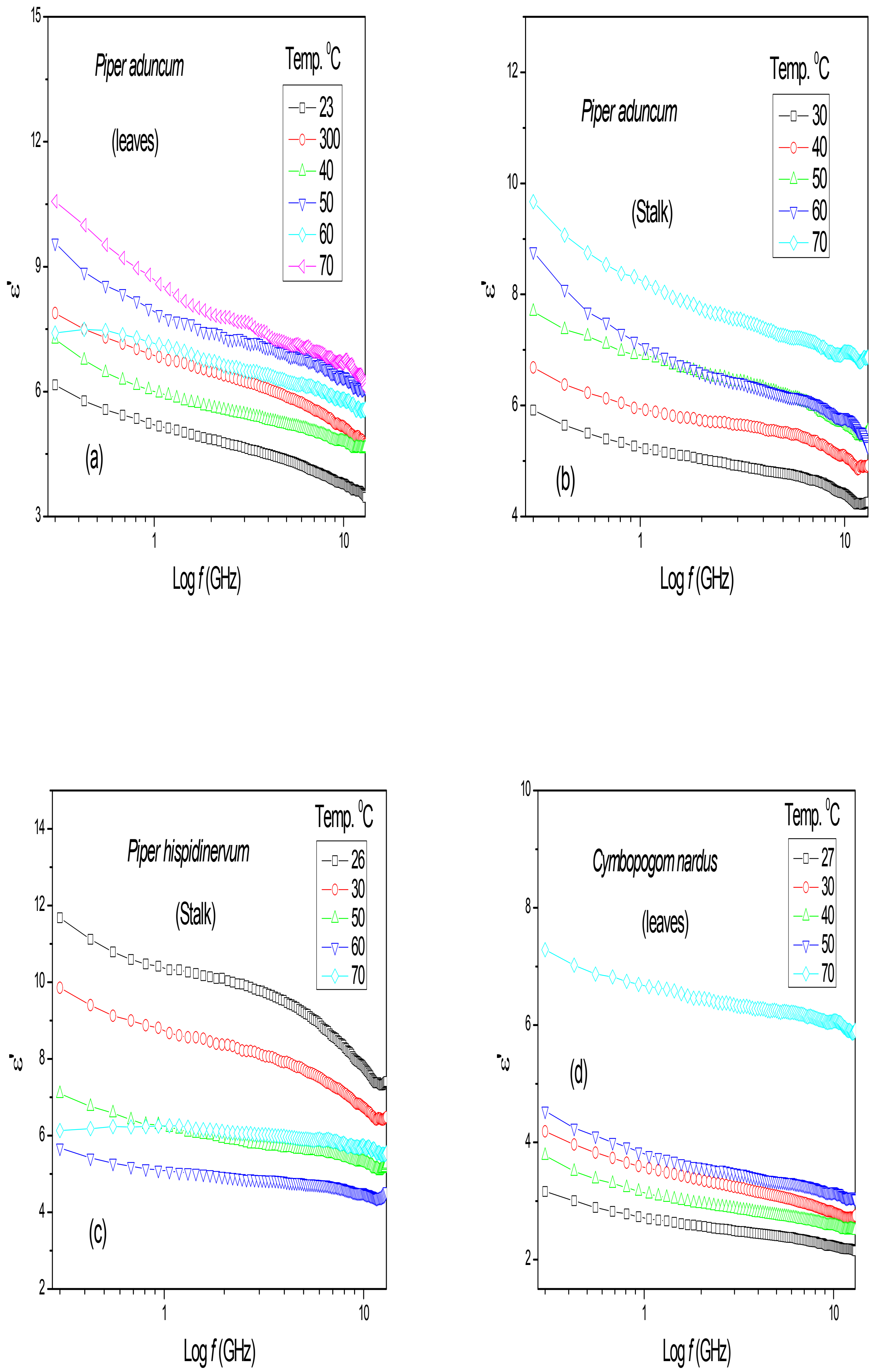

Figure S6. (a) – (d) Dielectric constant of the plant/water mixtures samples with 1:1 mass ratio as function of frequency and temperature.

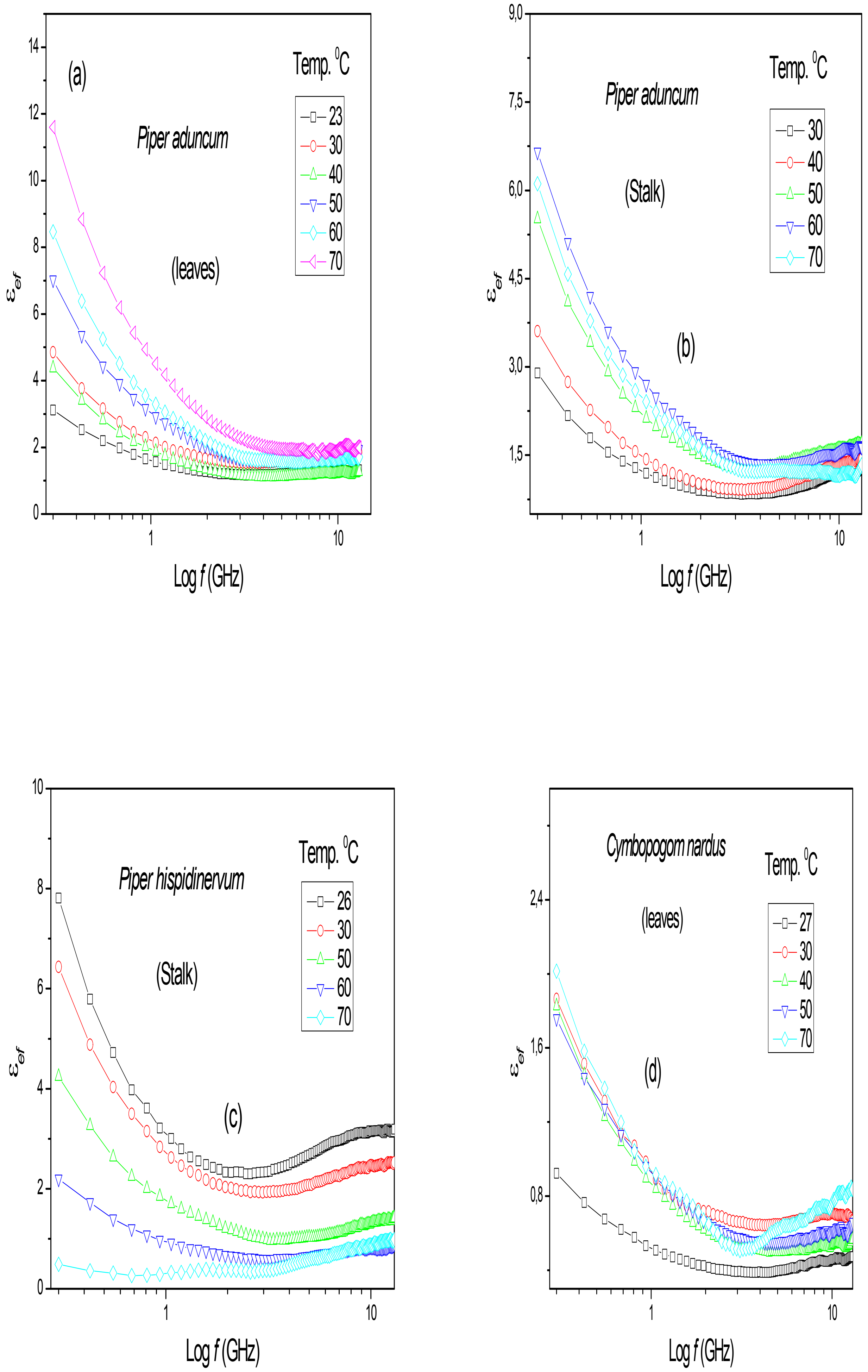

Figure S7. (a) – (d) Dielectric loss factor of the plant/water mixtures samples with 1:1 mass ratio as function of frequency and temperature.

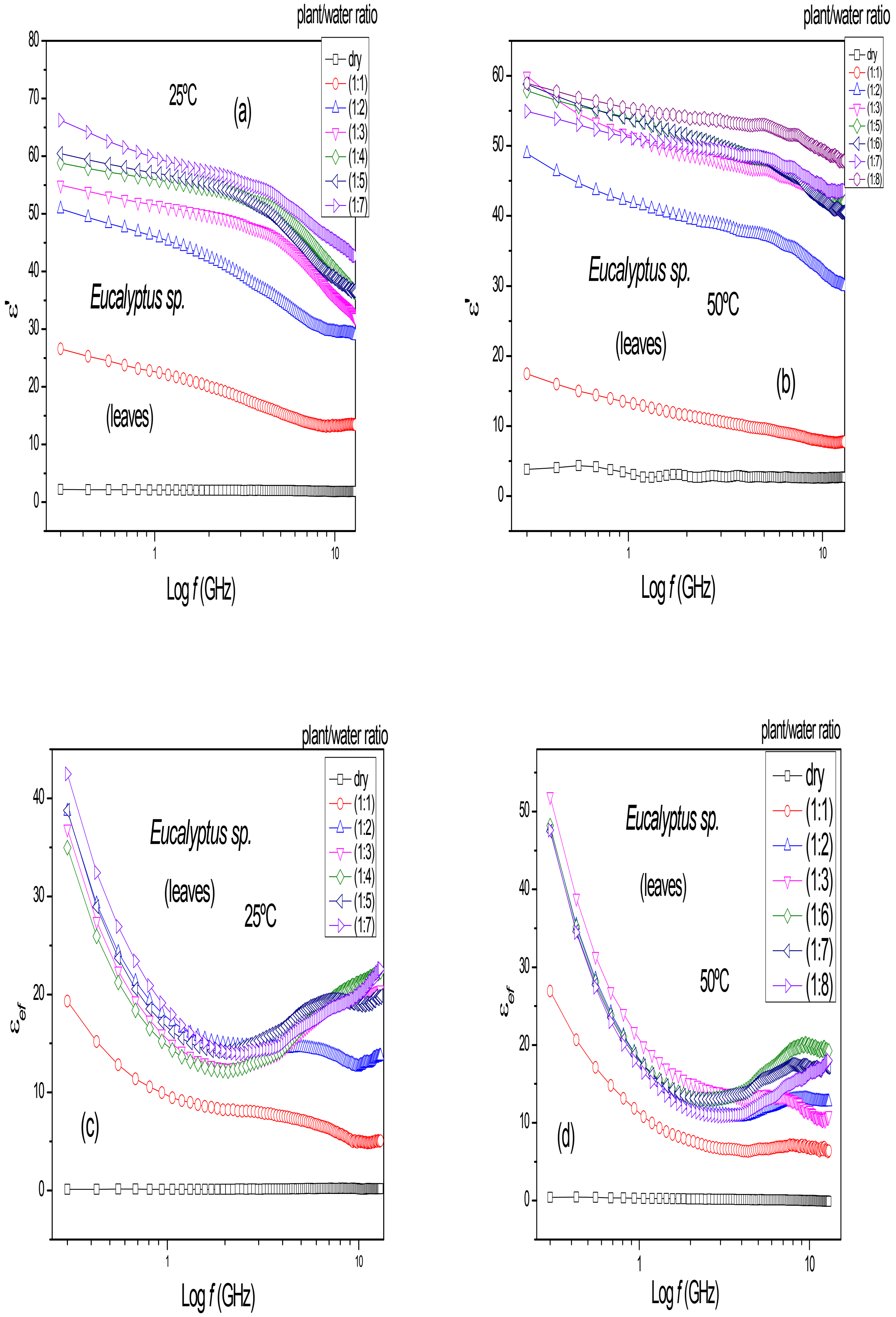

Figure S8. (a) – (b) Dielectric constant and (c) –(d) loss factor of the *Eucalyptus sp.*/water mixtures at different plant/water mass ratio and at two different temperatures as function of frequency. For comparison the results for dry plant are shown too.

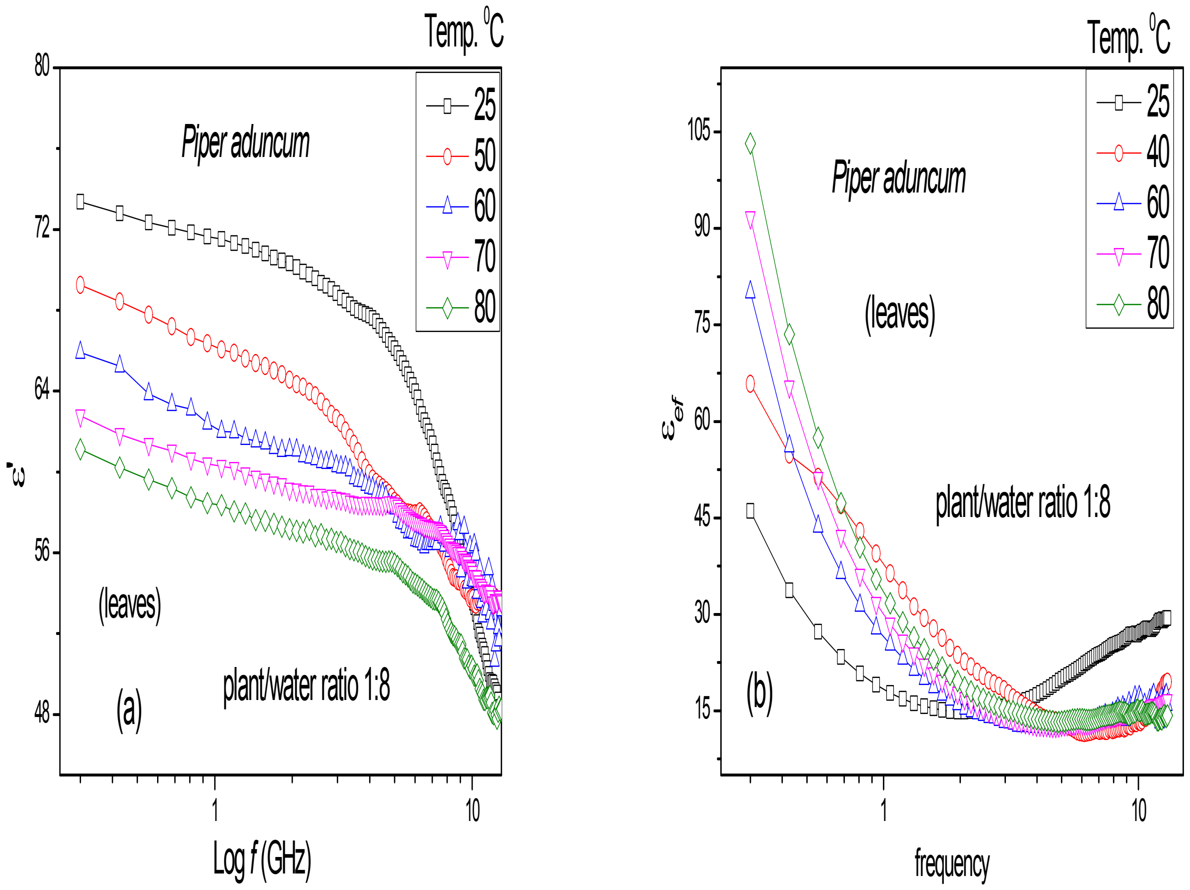

Figure S9. (a) Dielectric constant and (b) loss factor of the *Piper aduncum* /water mixtures at 1:8 plant/water mass ratio, as function of temperatures and frequency.

Table S1

	0.915 GHz					2.45 GHz			
Plant	T (°C)	ε'	ε_{ef}	Tg δ	Dp (cm)	ε'	ε_{ef}	Tg δ	Dp (cm)
Eucalyptus sp. (leaves)	27	1.519	0.042	0.027	30.53	1.491	0.038	0.026	12.33
	35	1.547	0.043	0.027	30.12	1.519	0.046	0.030	10.45
	40	1.606	0.058	0.036	22.83	1.578	0.053	0.033	9.25
	50	1.643	0.086	0.052	15.61	1.602	0.063	0.039	7.86
	70	1.661	0.068	0.040	19.85	1.611	0.057	0.035	8.63
P. Aduncum (leaves)	30	1.568	0.063	0.040	20.73	1.531	0.056	0.036	8.61
	35	1.611	0.071	0.044	18.63	1.565	0.067	0.043	7.20
	40	1.645	0.089	0.054	14.91	1.593	0.079	0.050	6.18
	50	1.588	0.086	0.054	15.18	1.538	0.077	0.050	6.27
	60	1.547	0.074	0.048	17.45	1.498	0.065	0.044	7.26
	70	1.588	0.094	0.059	13.89	1.531	0.077	0.051	6.20
P. Aduncum (stalk)	26	1.606	0.088	0.055	15.0	1.557	0.077	0.050	6.26
	35	1.601	0.101	0.063	12.97	1.544	0.089	0.058	5.39
	40	1.599	0.097	0.061	13.57	1.541	0.085	0.055	5.68
	60	1.612	0.115	0.071	11.51	1.551	0.089	0.057	5.41
	70	1.582	0.105	0.066	12.44	1.522	0.076	0.050	6.28
P. hispid. (leaves)	30	1.825	0.095	0.052	14.77	1.768	0.088	0.050	5.84
	35	1.7355	0.087	0.050	15.77	1.682	0.082	0.049	6.12
	40	1.7439	0.084	0.048	16.35	1.699	0.077	0.045	6.59
	50	1.7017	0.099	0.058	13.75	1.643	0.086	0.052	5.78
	60	1.813	0.104	0.057	13.42	1.746	0.102	0.058	5.02
	70	1.818	0.100	0.055	14.01	1.743	0.085	0.049	6.03
P. hispid. (stalk)	27	1.372	0.055	0.040	22.02	1.326	0.054	0.041	8.24
	35	1.558	0.0856	0.055	15.20	1.509	0.074	0.049	6.43
	50	1.574	0.109	0.069	11.93	1.521	0.088	0.058	5.43
	60	1.591	0.097	0.061	13.49	1.544	0.078	0.051	6.15
	70	1.603	0.087	0.054	15.15	1.551	0.076	0.049	6.38
Cymbop. nardus (leaves)	29	1.433	0.054	0.038	22.86	1.403	0.053	0.038	8.68
	35	1.549	0.065	0.042	19.79	1.508	0.059	0.039	8.01
	40	1.472	0.069	0.047	18.25	1.437	0.062	0.043	7.49
	50	1.555	0.090	0.058	14.37	1.495	0.079	0.053	5.98
	60	1.598	0.101	0.063	13.04	1.540	0.096	0.062	5.01
	70	1.539	0.102	0.066	12.69	1.476	0.088	0.060	5.35

Table S1. Dielectric properties of dry plants at various temperature at 0.915 GHz and 2.45GHz.

Table S2

	0.915 GHz					2.45 GHz			
Plant	T (°C)	ε'	ε_{ef}	Tg δ	Dp (cm)	ε'	ε_{ef}	Tg δ	Dp (cm)
Eucalyptus sp. (leaves)	24	3.556	0.560	0.157	3.52	3.373	0.426	0.126	1.68
	30	7.331	2.078	0.283	1.37	6.874	1.287	0.187	0.79
	40	7.994	2.014	0.252	1.47	7.544	1.188	0.157	0.90
	50	9.223	3.299	0.357	0.97	8.503	1.840	0.216	0.62
	60	9.186	4.383	0.477	0.74	8.348	2.374	0.284	0.48
	70	9.898	5.919	0.598	0.57	9.285	2.986	0.321	0.40
P. Aduncum (leaves)	23	5.258	1.668	0.317	1.45	4.746	1.206	0.254	0.71
	30	6.923	2.322	0.335	1.19	6.369	1.594	0.250	0.62
	40	6.060	2.042	0.337	1.27	5.535	1.236	0.223	0.74
	50	8.028	3.213	0.400	0.94	7.264	1.794	0.247	0.59
	60	7.208	3.617	0.501	0.79	6.592	1.895	0.287	0.53
	70	8.827	5.012	0.567	0.64	7.778	2.508	0.322	0.44
P. Aduncum (stalk)	30	5.277	1.304	0.247	1.85	4.981	0.859	0.172	1.01
	40	5.972	1.585	0.265	1.62	5.700	0.942	0.165	0.98
	50	6.925	2.346	0.338	1.18	6.512	1.371	0.210	0.73
	60	7.180	2.964	0.413	0.96	6.504	1.566	0.241	0.64
	70	8.327	2.642	0.317	1.15	7.648	1.413	0.185	0.76
P. hispid. (leaves)	28	5.235	1.728	0.330	1.40	4.765	1.151	0.241	0.74
	30	5.375	1.656	0.308	1.47	4.936	1.094	0.221	0.79
	40	11.447	7.313	0.639	0.50	10.755	4.086	0.380	0.32
	50	11.643	7.973	0.685	0.47	10.901	4.012	0.368	0.32
	60	12.849	10.442	0.813	0.38	12.229	5.189	0.424	0.27
	70	11.051	12.373	1.119	0.31	11.271	5.910	0.524	0.22
P. hispid. (stalk)	26	10.427	3.272	0.314	1.04	9.948	2.296	0.231	0.54
	30	8.820	2.887	0.327	1.08	8.222	1.965	0.239	0.57
	50	6.284	1.870	0.297	1.41	5.842	1.072	0.183	0.88
	60	5.106	0.990	0.194	2.39	4.885	0.606	0.124	1.42
	70	6.248	0.284	0.045	9.17	6.054	0.354	0.058	2.70
Cymbop. nardus (leaves)	27	2.738	0.538	0.196	3.21	2.527	0.403	0.159	1.54
	30	3.605	0.999	0.277	2.00	3.301	0.689	0.209	1.03
	40	3.176	0.911	0.287	2.06	2.904	0.568	0.196	1.17
	50	3.852	0.973	0.252	2.12	3.521	0.615	0.175	1.19
	70	6.701	0.973	0.145	2.78	6.383	0.558	0.087	1.76

Table S2. Dielectric behavior of plants with addition of water (1:1) as function of temperature at 0.915 GHz and 2.45GHz.

Table S3

	0.915 GHz					2.45 GHz			
Plant	T (°C)	ε'	ε_{ef}	Tg δ	Dp (cm)	ε'	ε_{ef}	Tg δ	Dp (cm)
P. Aduncum (leaves)	25	71.669	19.308	0.269	4.61	69.587	15.057	0.216	2.17
	30	42.445	33.247	0.783	2.17	51.350	16.047	0.312	1.76
	40	44.519	39.971	0.898	1.88	55.024	20.401	0.371	1.43
	50	66.414	28.357	0.427	3.06	63.792	17.248	0.270	1.82
	60	62.521	28.387	0.454	2.97	60.661	14.418	0.238	2.12
	70	60.437	32.398	0.536	2.58	58.850	15.394	0.262	1.96
	80	58.508	36.224	0.619	2.29	56.832	16.880	0.297	1.76
	90	34.977	51.116	1.461	1.42	49.824	24.240	0.486	1.16

Table S3. Dielectric parameters of *Piper aduncum* with water (1:8) as function of temperature at 0.915 GHz and 2.45GHz.

Table S4

	915 MHz					2.45 GHz			
Plant/ water ratio	**T (°C)**	**ε'**	**ε''**	**Tg δ**	**Dp** (mm)	**ε'**	**ε''**	**Tg δ**	**Dp** (mm)
Dry plant	25	2.138	0.119	0.056	127.752	2.063	0.140	0.068	40.091
	50	2.109	0.331	0.157	45.961	1.959	0.175	0.089	31.192
	70	1.890	0.157	0.083	91.654	1.847	0.072	0.039	73.341
	80	3.484	1.107	0.318	17.798	2.736	0.759	0.277	8.572
(1:1)	25	22.842	10.060	0.440	5.068	19.169	8.075	0.421	2.156
	50	22.911	12.061	0.526	4.271	20.560	7.061	0.343	2.537
	70	17.541	9.464	0.540	4.770	15.772	5.575	0.354	2.816
	80	13.581	8.479	0.624	4.731	11.073	5.082	0.459	2.613
(1:2)	25	46.407	18.287	0.394	3.957	41.034	14.742	0.359	1.719
	50	42.543	19.42	0.457	3.588	39.949	11.350	0.284	2.19
	70	35.739	20.380	0.570	3.173	32.472	11.129	0.343	2.023
	80	42.327	25.264	0.597	2.794	39.000	13.194	0.338	1.869
(1:3)	25	51.746	16.185	0.313	4.690	49.214	12.605	0.256	2.185
	50	47.521	22.092	0.465	3.337	43.037	14.354	0.333	1.804
	70	43.173	20.070	0.465	3.501	40.877	11.740	0.287	2.142
	80	51.917	31.048	0.598	2.518	48.489	15.758	0.325	1.7432
(1:4)	25	56.145	15.424	0.275	5.113	53.853	12.495	0.232	2.303
	50	52.916	18.896	0.357	4.076	51.024	11.521	0.226	2.430
	70	49.306	23.677	0.480	3.176	47.236	12.986	0.275	2.080
	80	5.018	0.602	0.120	3.887	4.935	0.396	0.080	2.185
(1:5)	25	57.295	17.376	0.303	4.594	54.163	14.578	0.269	1.984
	50	19.845	1.215	0.061	3.824	18.794	2.668	0.142	6.345
	70	49.150	22.481	0.457	3.332	47.030	12.596	0.268	2.139
	80	54.272	25.945	0.478	3.040	50.639	14.422	0.285	1.941
(1:6)	25	---------	---------	---------	---------	---------	---------	--------	---------
	50	58.911	17.615	0.299	4.594	57.279	12.951	0.226	2.290
	70	13.203	6.860	0.52	5.522	13.122	12.07	0.92	1.169
	80	53.702	24.360	0.454	3.213	50.813	15.267	0.300	1.839
(1:7)	25	60.096	19.426	0.323	4.214	56.652	13.939	0.246	2.119
	50	61.497	19.078	0.310	4.337	59.413	13.019	0.219	2.320
	70	65.748	22.555	0.343	3.803	64.091	13.199	0.206	2.375
	80	51.451	19.210	0.373	3.959	49.659	10.500	0.211	2.628
(1:8)	25	30.842	15.177	0.492	3.924	34.733	2.874	0.083	7.995
	50	57.792	18.320	0.317	4.380	56.293	11.081	0.197	2.650
	70	54.342	22.505	0.414	3.486	52.929	11.842	0.224	2.408
	80	55.606	25.488	0.458	3.127	53.955	12.449	0.231	2.313

Table S4. Dielectric parameters of *Eucalyptus sp.* with water as function of temperature at 0.915 GHz and 2.45GHz at various plant/water ratio

II. Diameters of the dielectric samples in cylindrical cavities

1. Dielectric properties of pure liquids, ethanol, methanol and glycerin as dielectric samples.

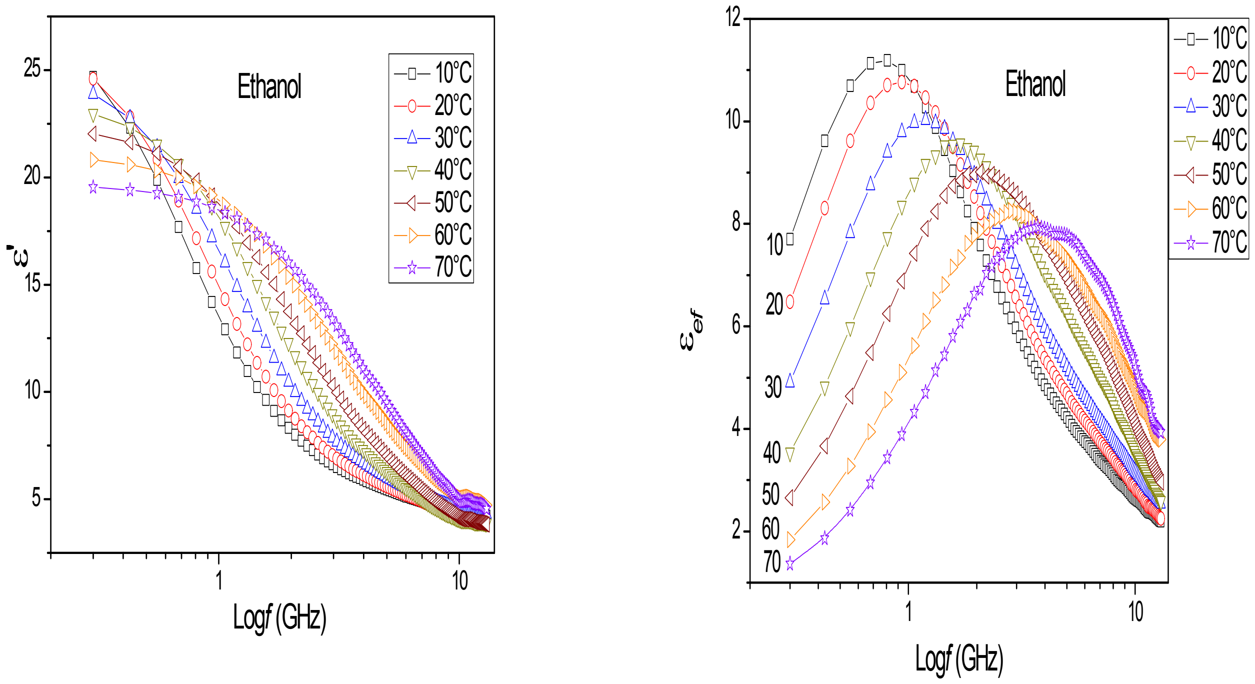

Figure S10. (a) Dielectric constant and (b) loss factor of ethanol as function of frequency and temperature.

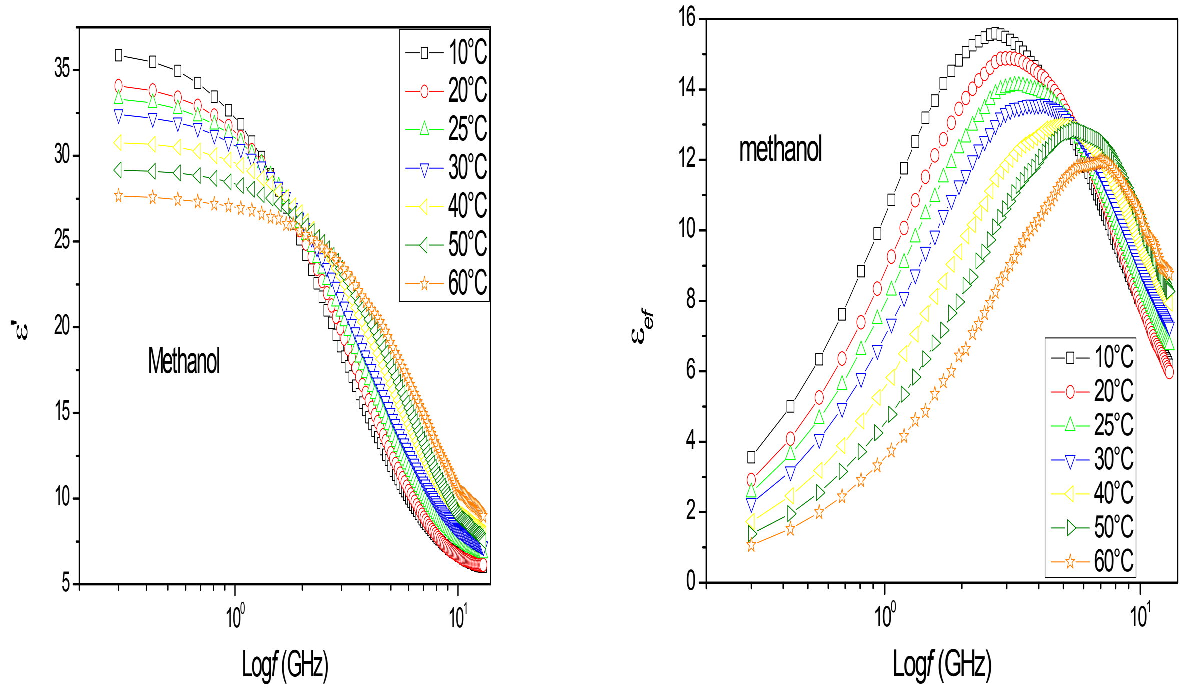

Figure 11. (a) Dielectric constant and (b) loss factor of methanol as function of frequency and temperature.

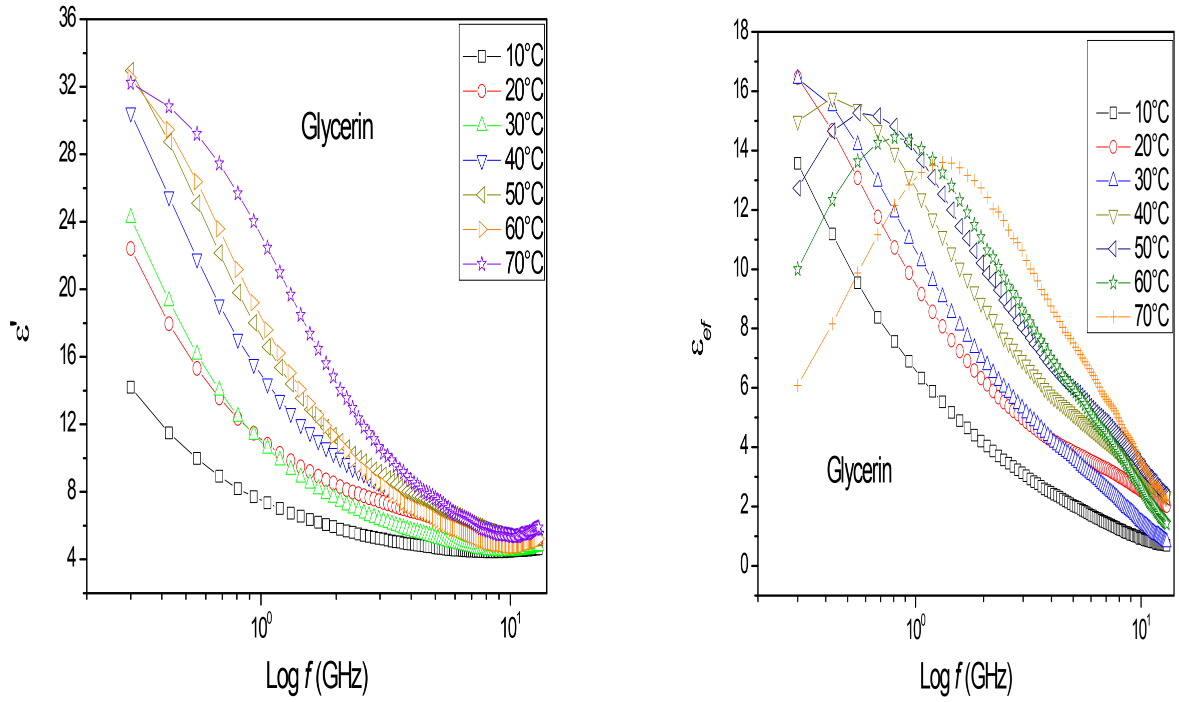

Figure S12. (a) Dielectric constant and (b) loss factor of glycerin as function of frequency and temperature.

2. Diameters of the dielectric samples in cylindrical cavities calculate from dielectric properties of pure liquids, ethanol, methanol and glycerin as dielectric samples.

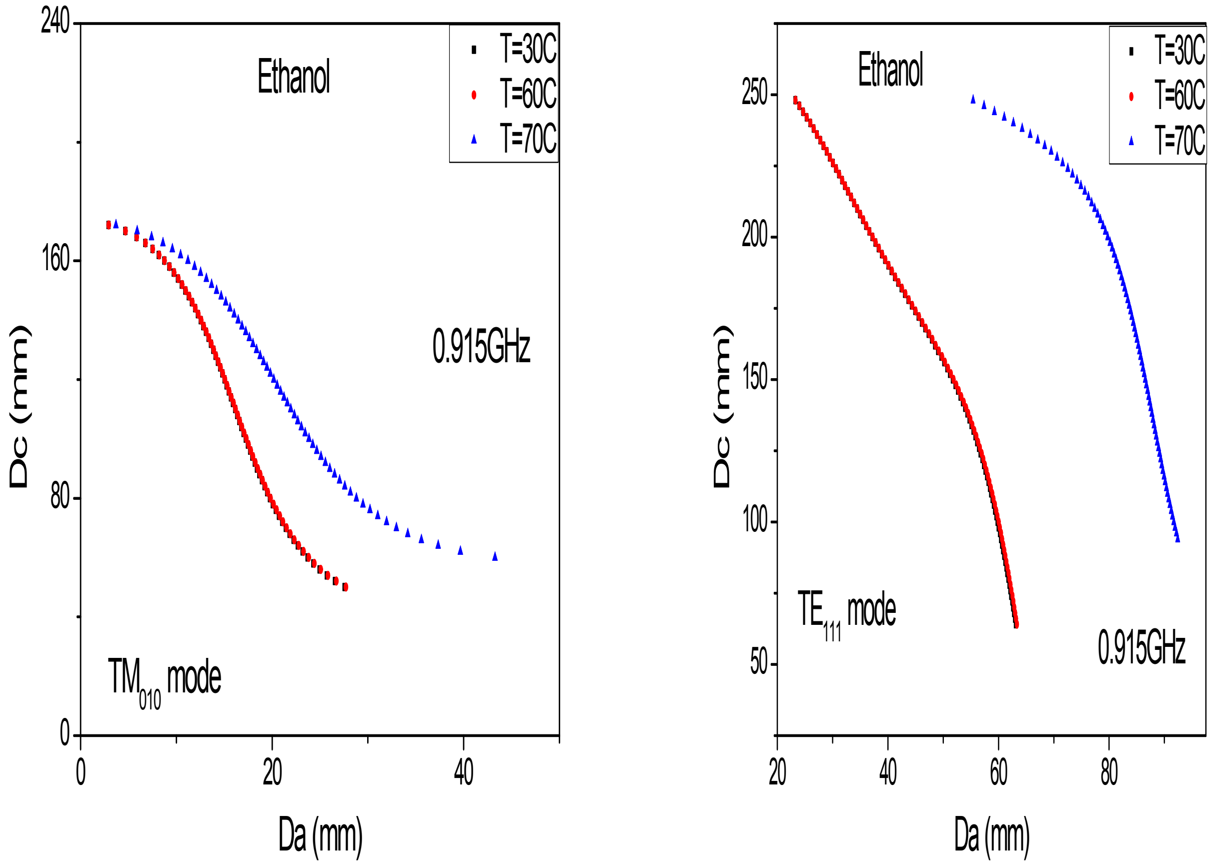

Figure S13. Internal diameter of a TM_{010} and TE_{111} cavities (Dc) as a function of the diameter (Da) of dielectric for the pure ethanol at two different temperatures for 0.915 GHz.

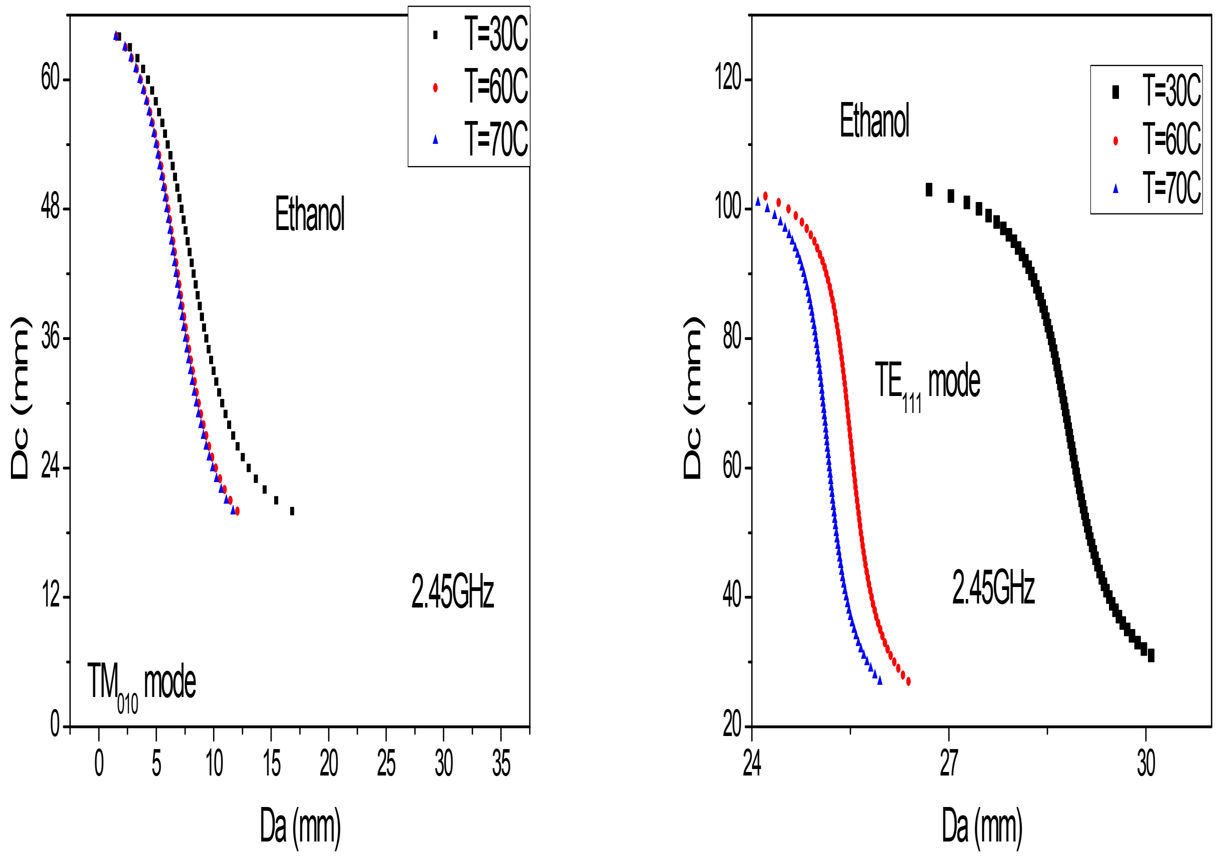

Figure S14. Internal diameter of a TM_{010} and TE_{111} cavities (Dc) as a function of the diameter (Da) of dielectric for the pure ethanol at two different temperatures for 2.45 GHz.

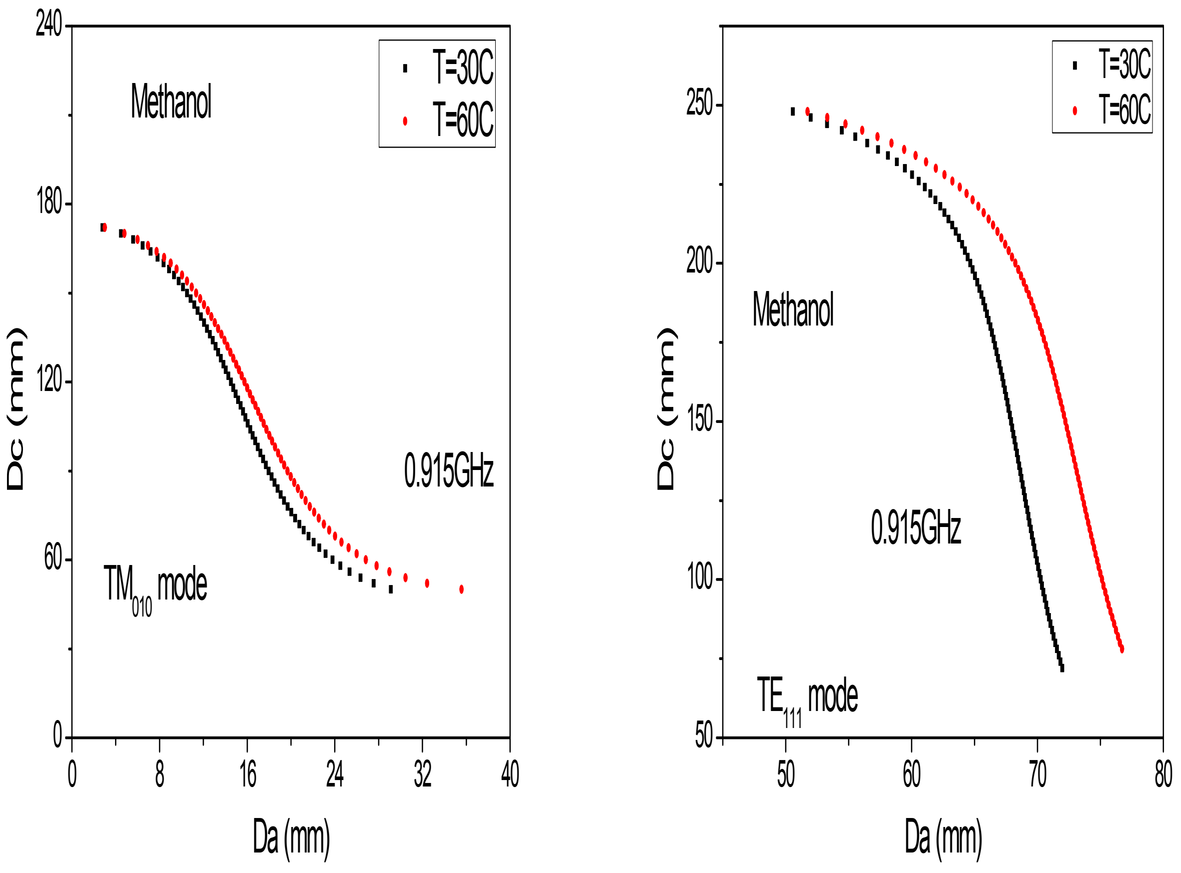

Figure S15. Internal diameter of a TM_{010} and TE_{111} cavities (Dc) as a function of the diameter (Da) of dielectric for the pure methanol at two different temperatures for 0.915 GHz.

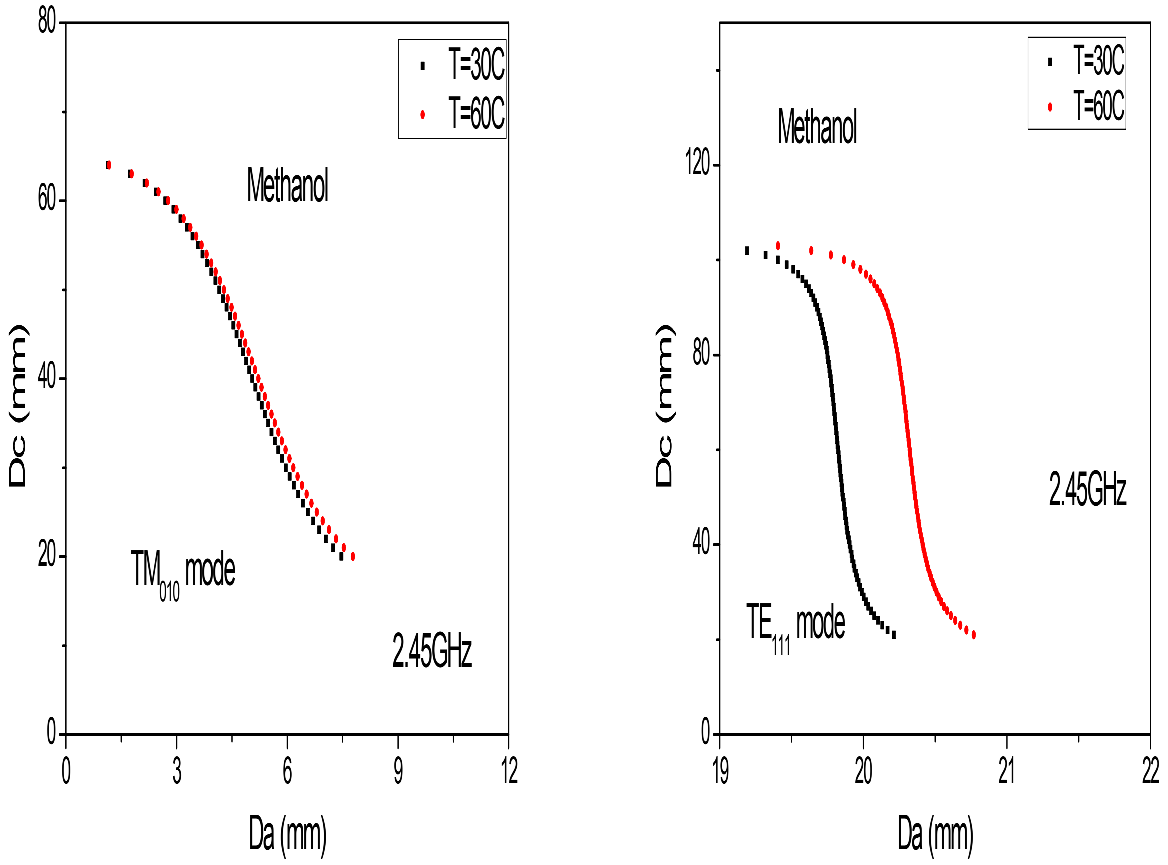

Figure S16. Internal diameter of a TM_{010} and TE_{111} cavities (Dc) as a function of the diameter (Da) of dielectric for the pure methanol at two different temperatures for 2.45 GHz.

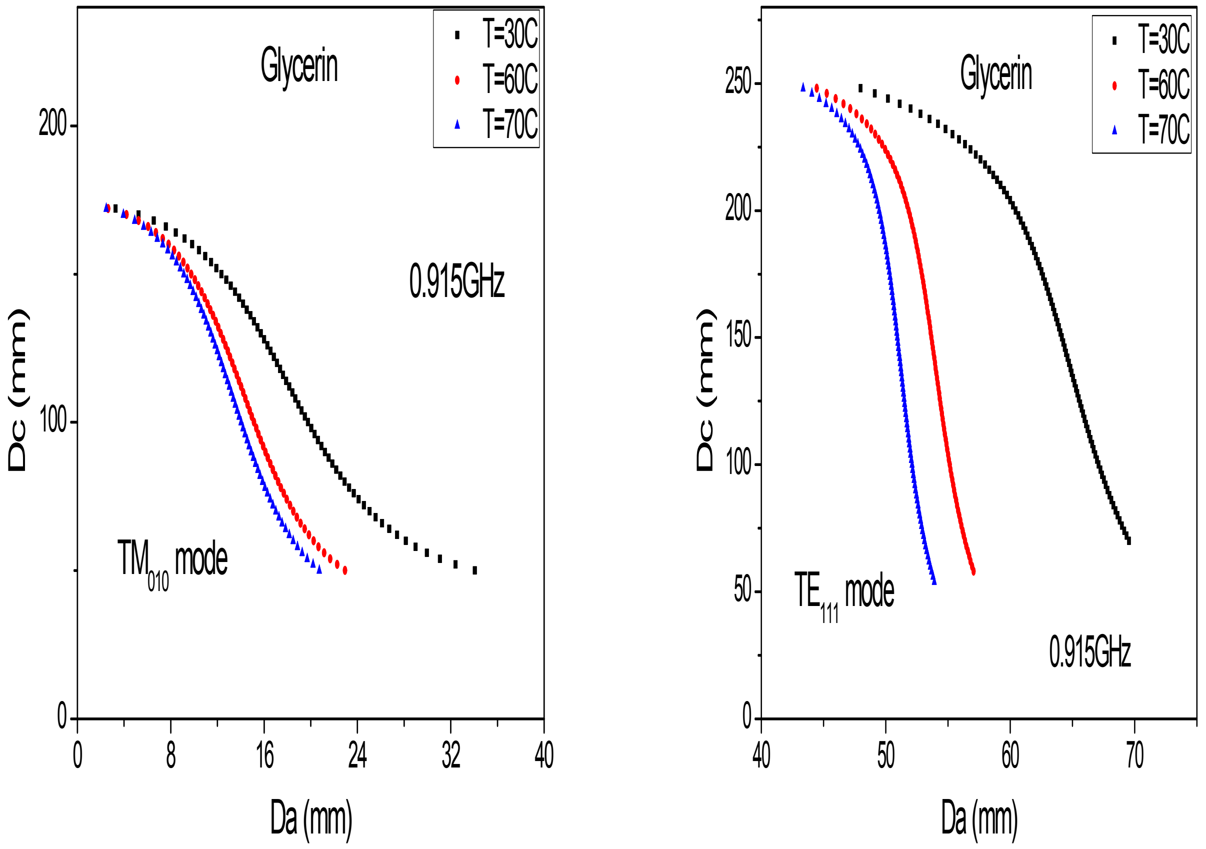

Figure S17. Internal diameter of a TM_{010} and TE_{111} cavities (Dc) as a function of the diameter (Da) of dielectric for the pure glycerin at two different temperatures for 0.915 GHz.

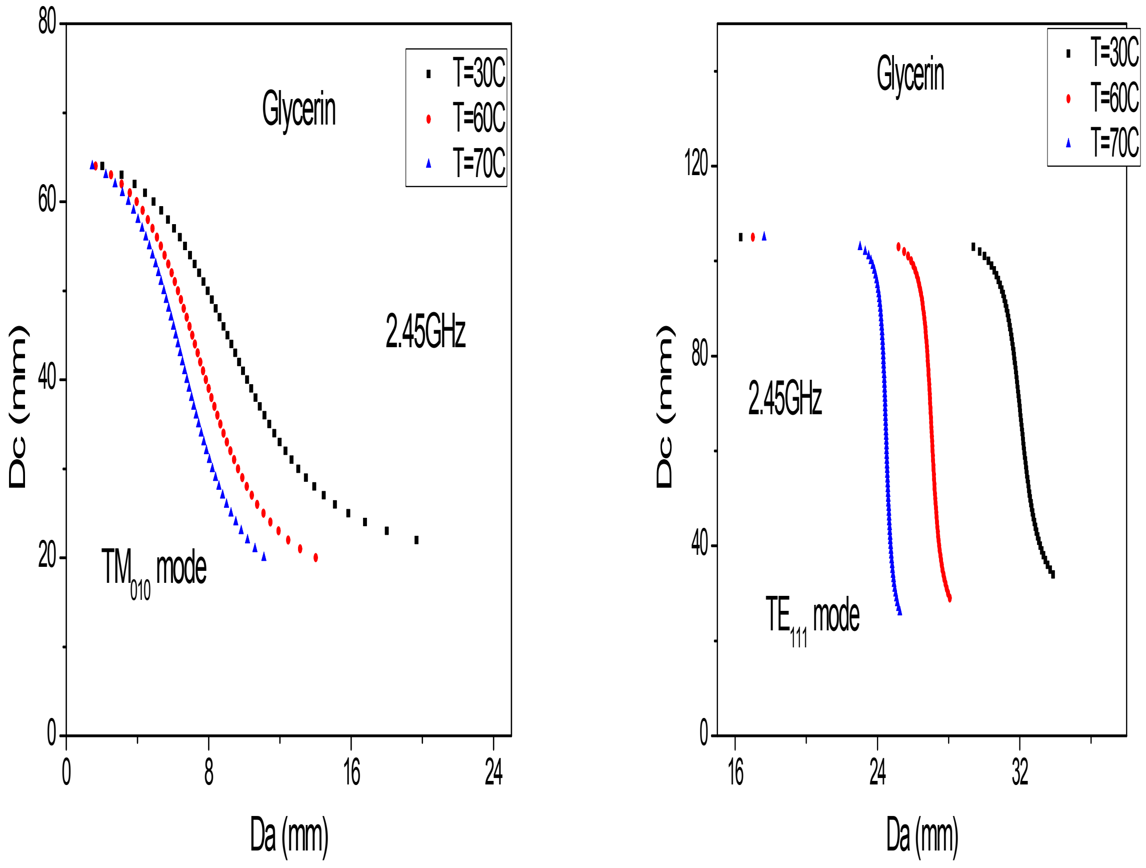

Figure S18. Internal diameter of a TM_{010} and TE_{111} cavities (Dc) as a function of the diameter (Da) of dielectric for the pure glycerin at two different temperatures for 2.45 GHz.

3. Diameters of the dielectric samples in cylindrical cavities calculate from dielectric properties of plant/water mixtures as dielectric samples.

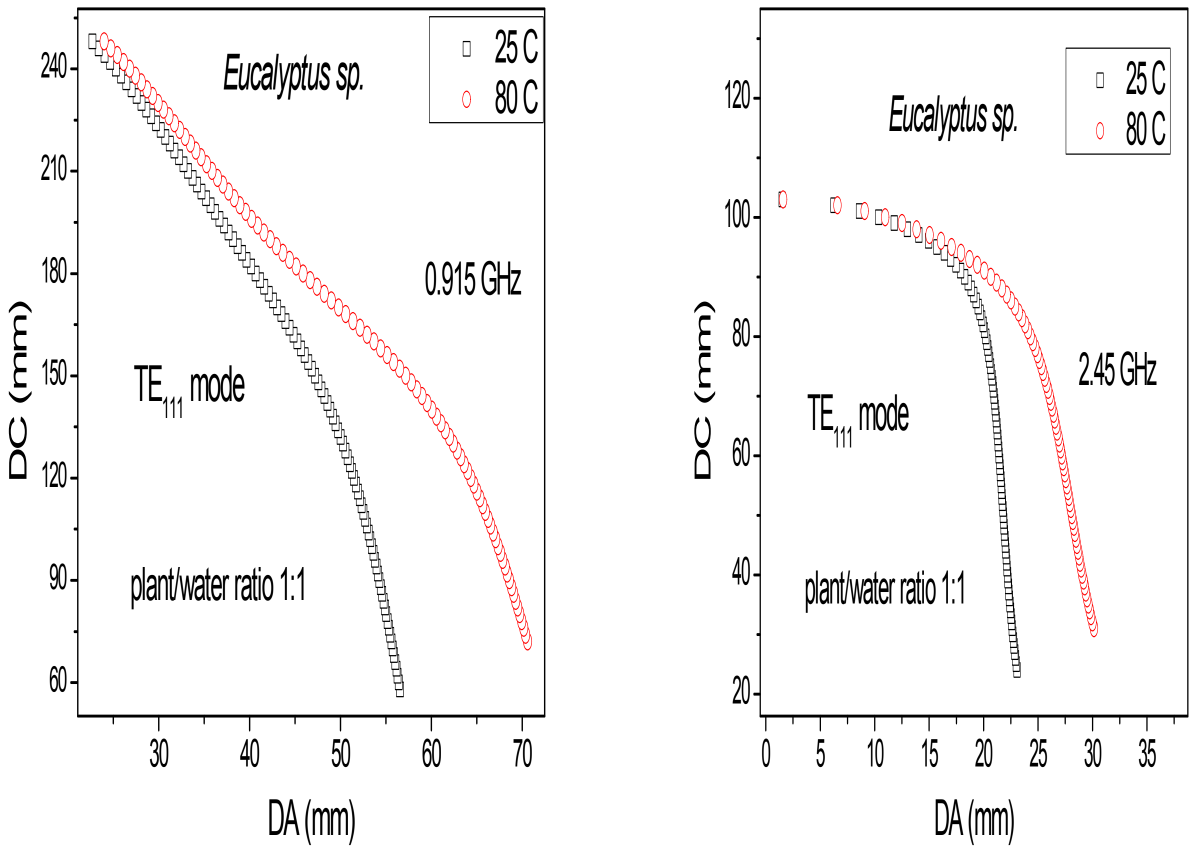

Figure S19. Internal diameter of a TE_{111} cavity (Dc) as a function of the diameter (Da) of the dielectric for the *Eucalyptus sp.*/water mixtures with 1:1 plant/water mass ratio at two different temperatures for 0.915 GHz and 2.45 GHz

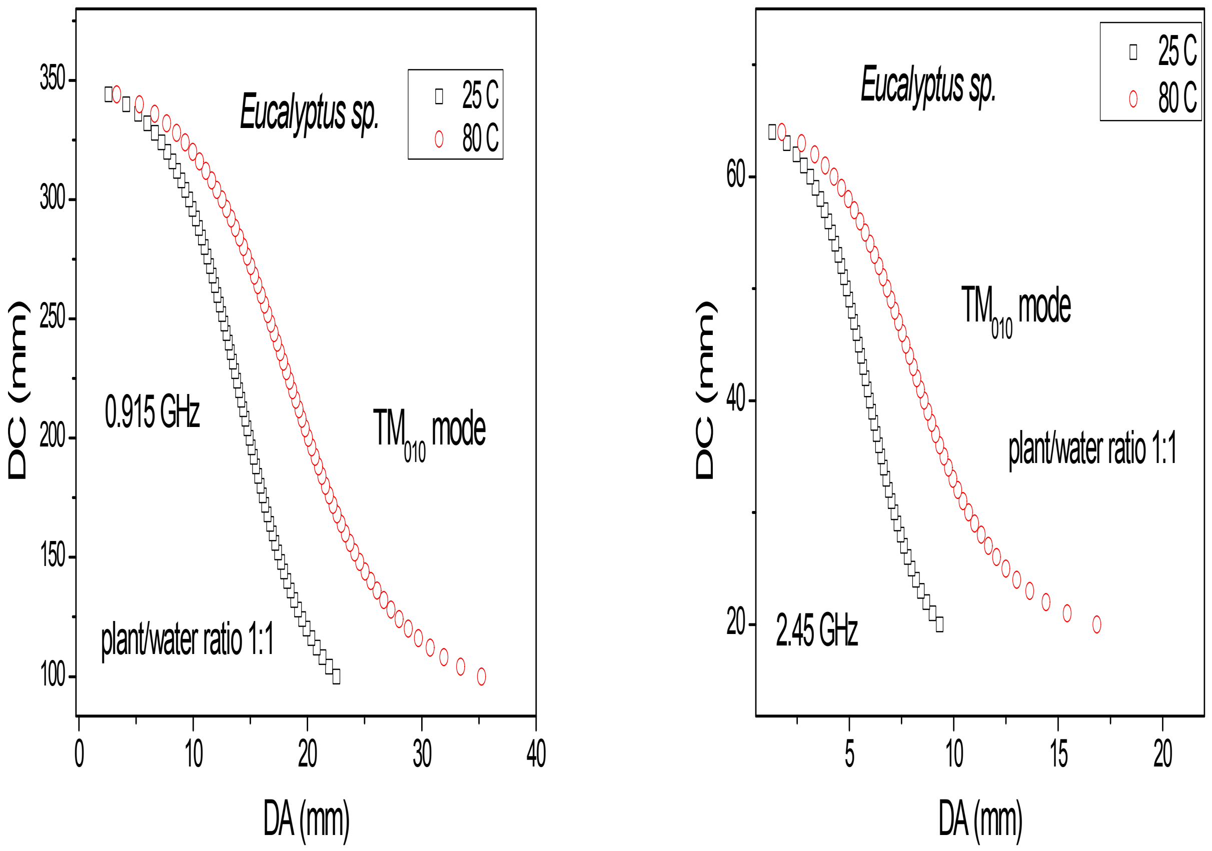

Figure S20. Internal diameter of a TM_{010} cavity (Dc) as a function of the diameter (Da) of the dielectric for the *Eucalyptus sp.*/water mixtures with 1:1 plant/water mass ratio at two different temperatures for 0.915 GHz and 2.45 GHz

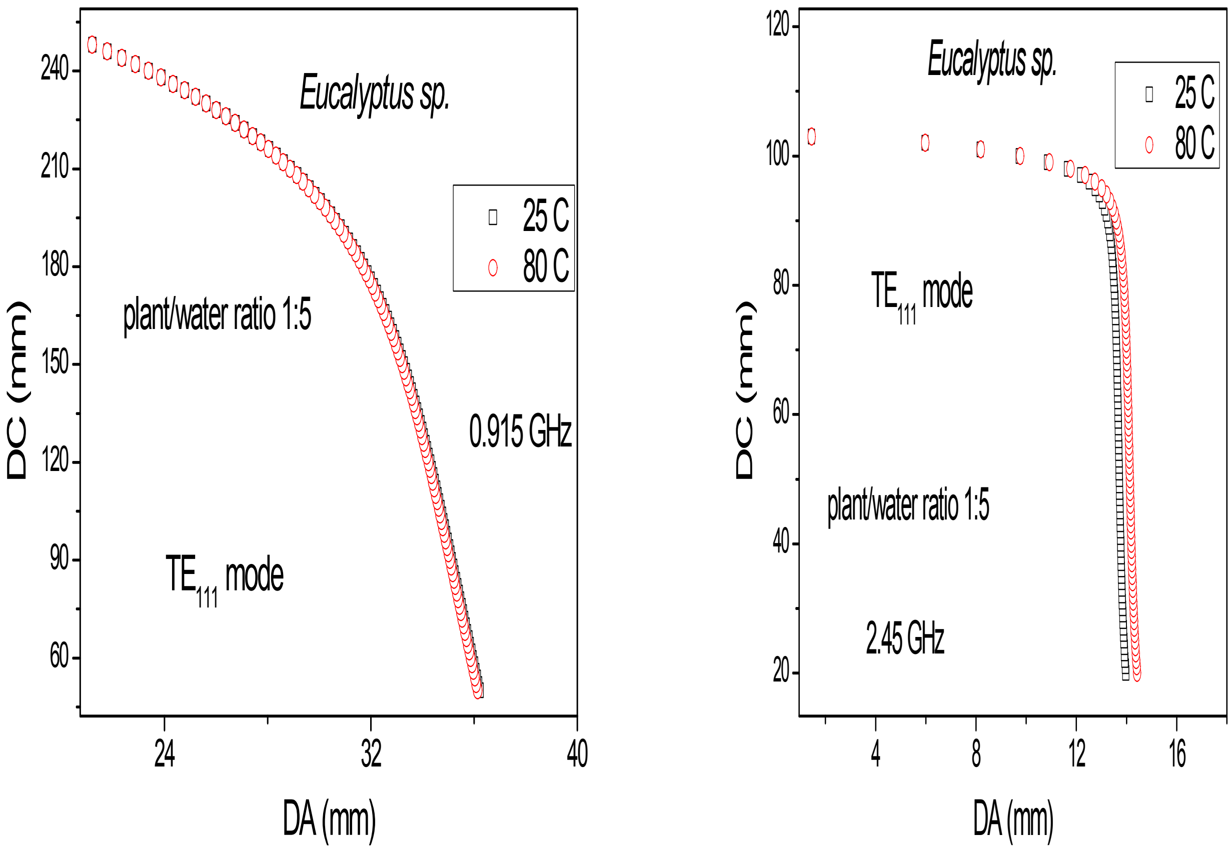

Figure S21. Internal diameter of a TE_{111} cavity (Dc) as a function of the diameter (Da) of the dielectric for the *Eucalyptus sp.*/water mixtures with 1:5 plant/water mass ratio at two different temperatures for 0.915 GHz and 2.45 GHz

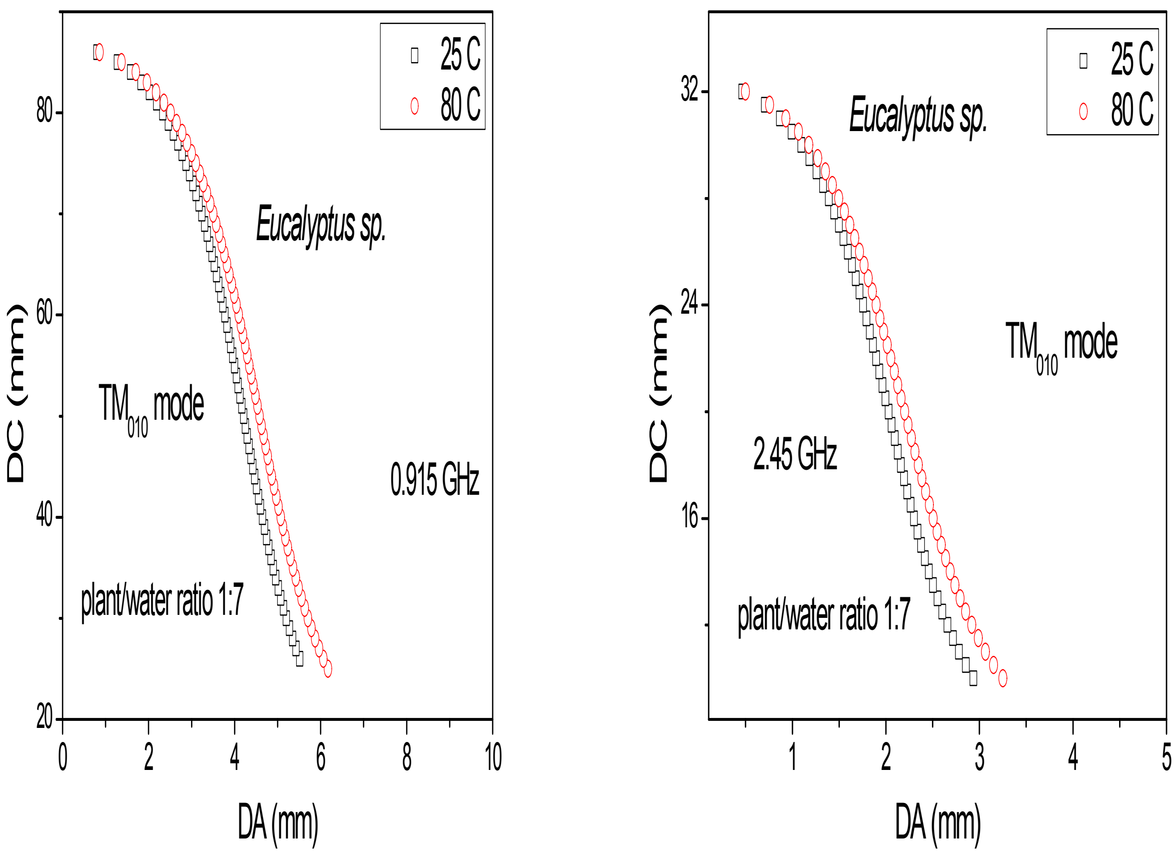

Figure S22. Internal diameter of a TM_{010} cavity (Dc) as a function of the diameter (Da) of the dielectric for the *Eucalyptus sp.*/water mixtures with 1:5 plant/water mass ratio at two different temperatures for 0.915 GHz and 2.45 GHz

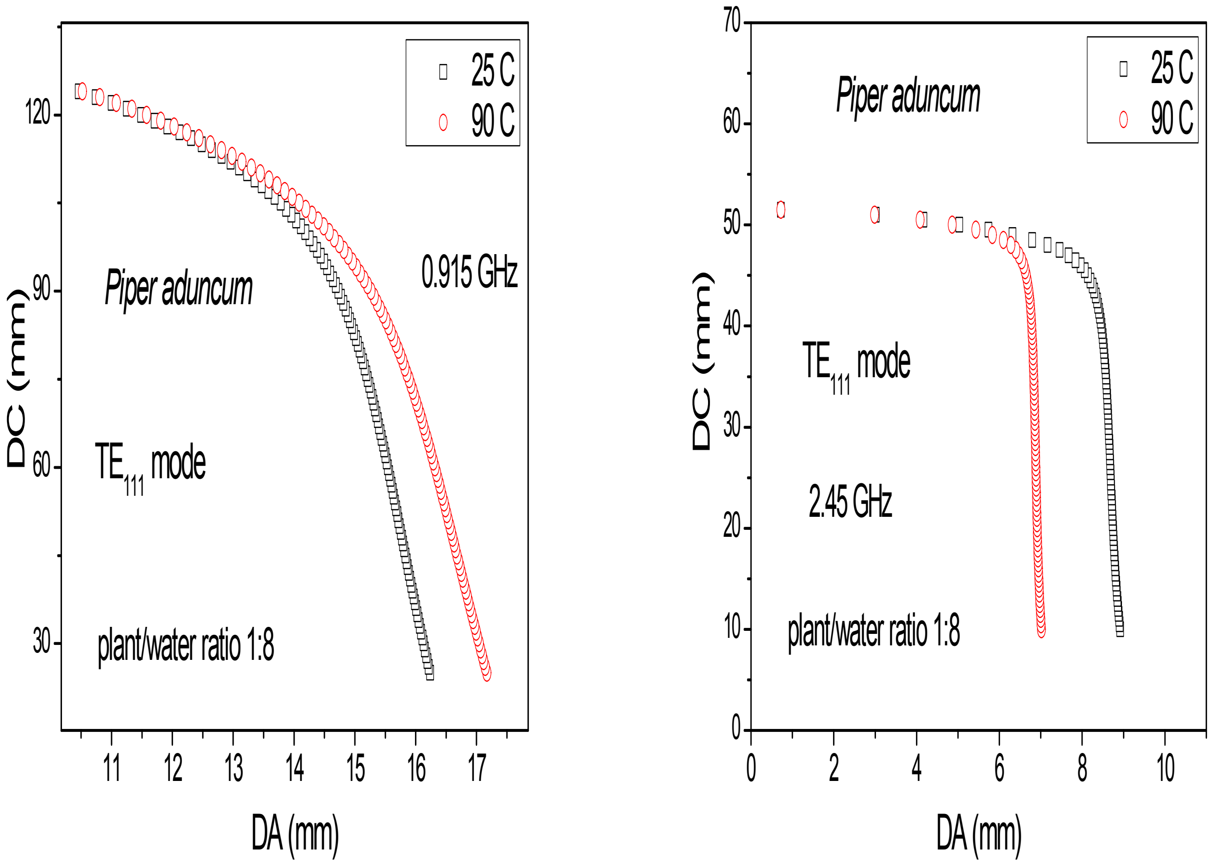

Figure S23. Internal diameter of a TE_{111} cavity (Dc) as a function of the diameter (Da) of the dielectric for the *Piper aduncum*/water mixtures with 1:8 plant/water mass ratio at two different temperatures for 0.915 GHz and 2.45 GHz.

III. Chromatograms of the Essentials oils extracted by MAE

1. Chromatograms of the experiments of table 1

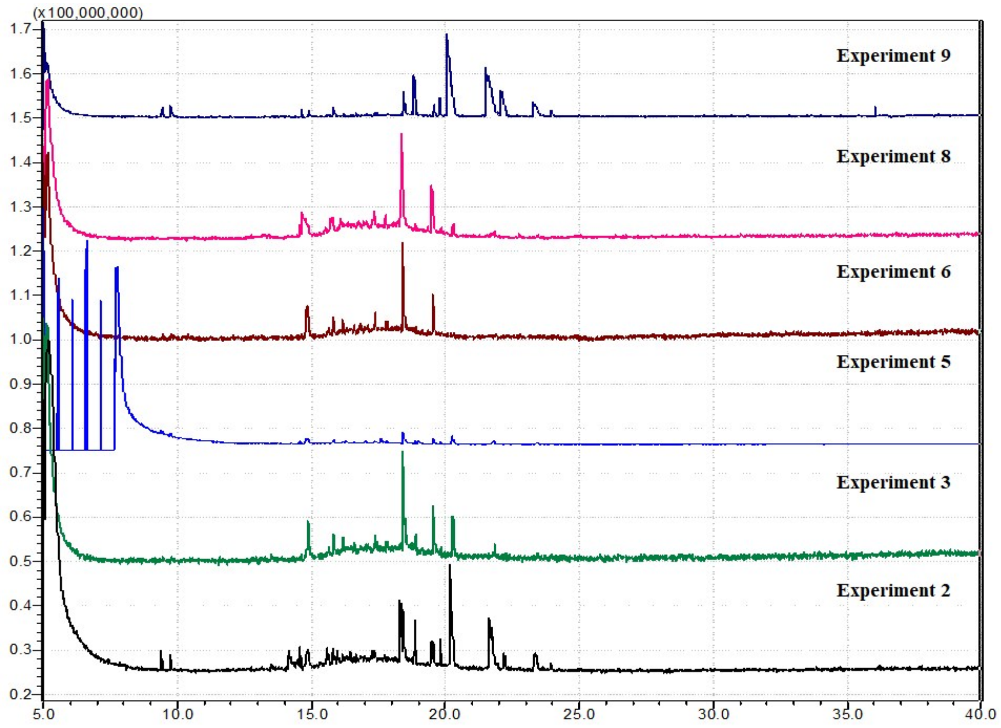

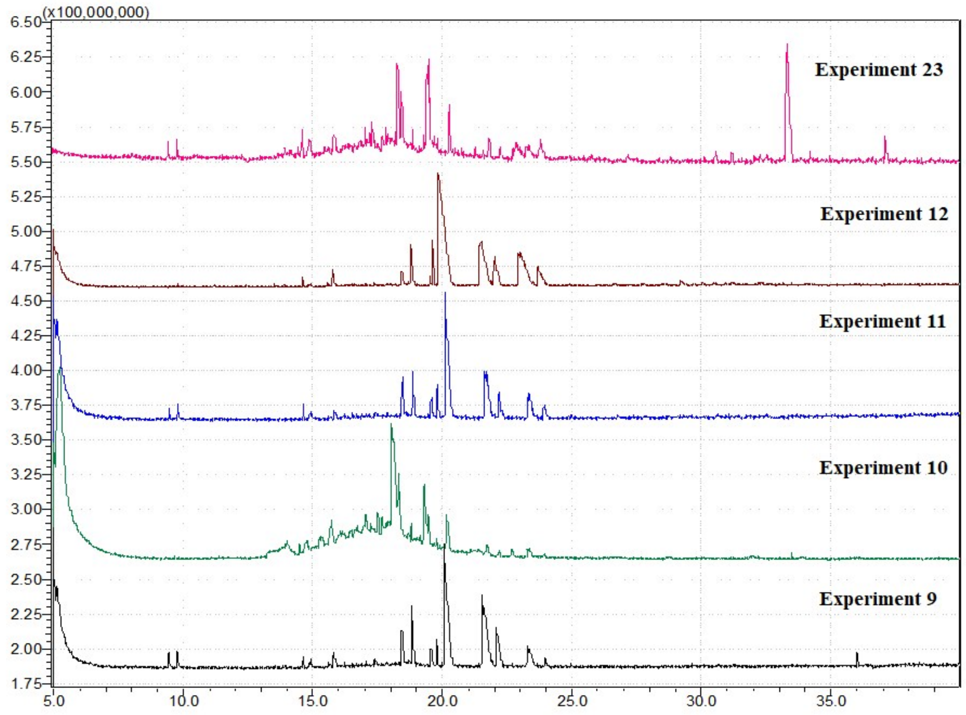

2. Chromatograms of the experiments of table 2

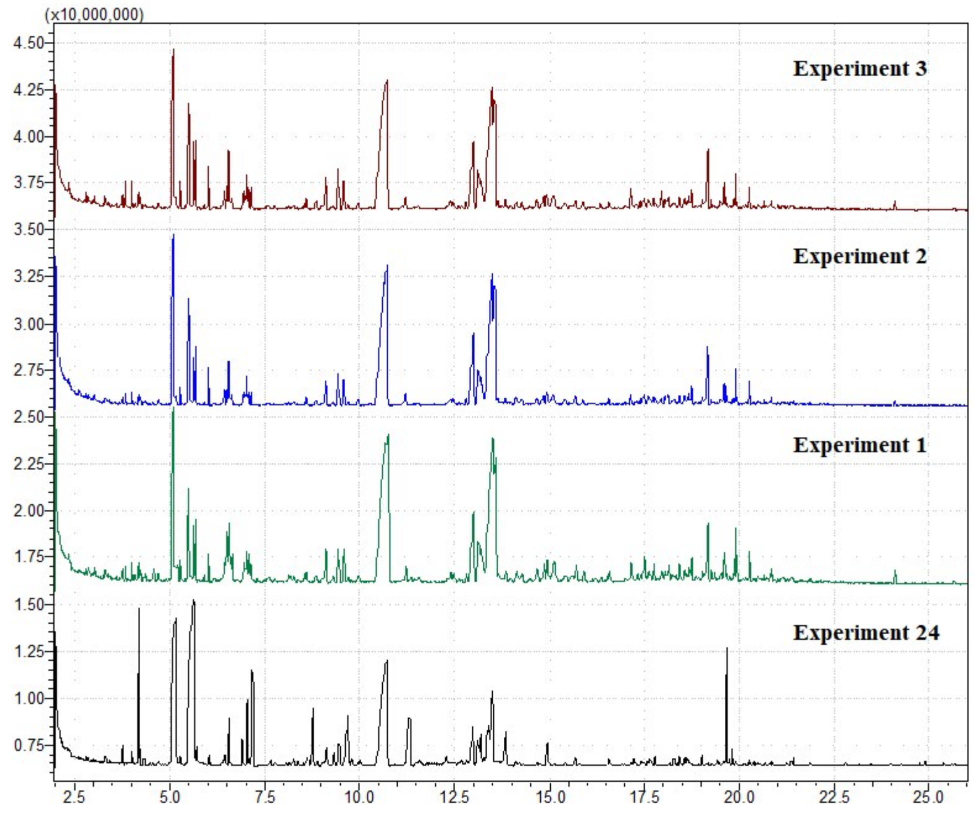

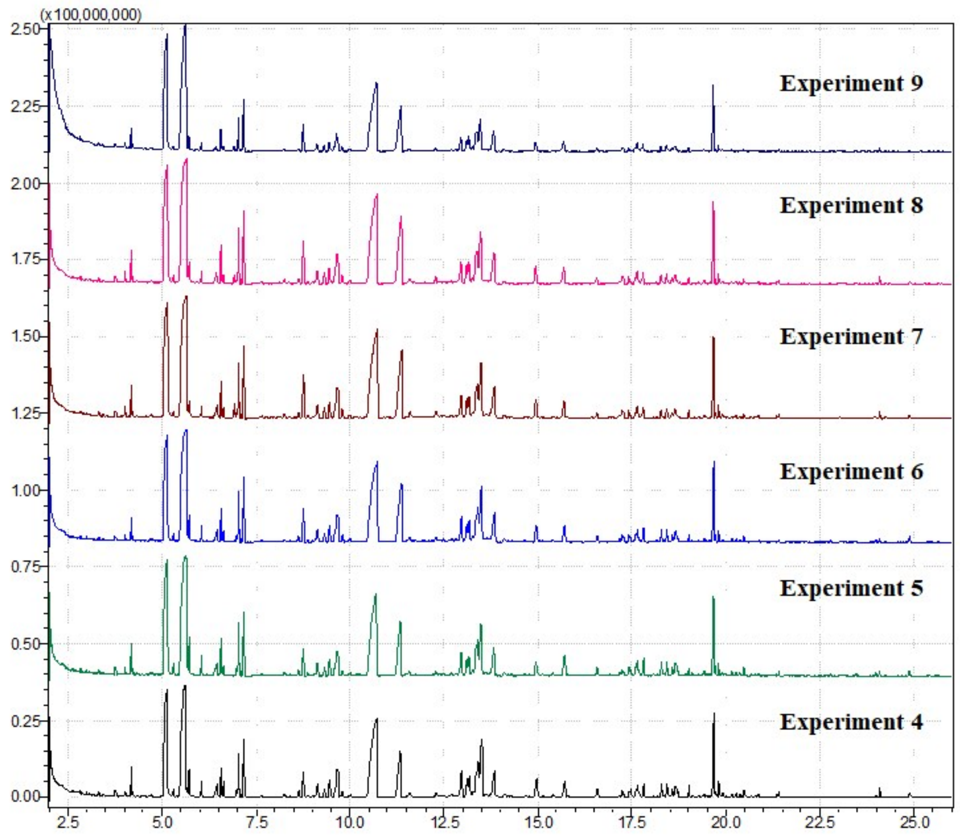

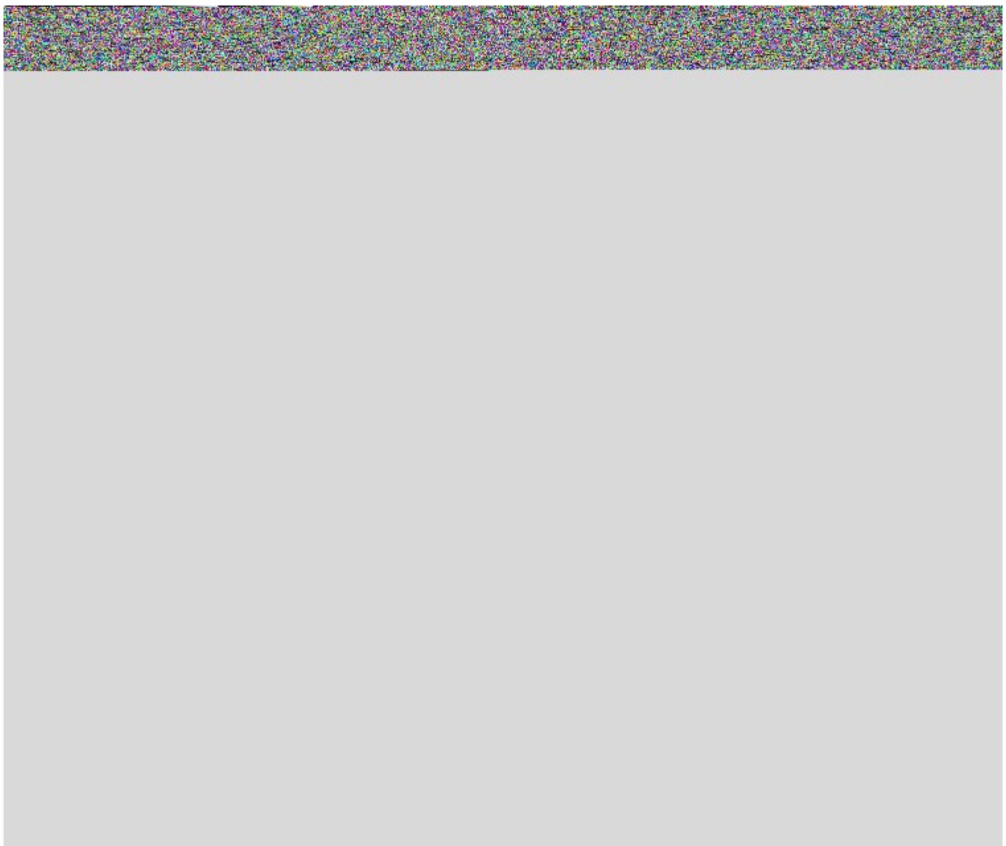

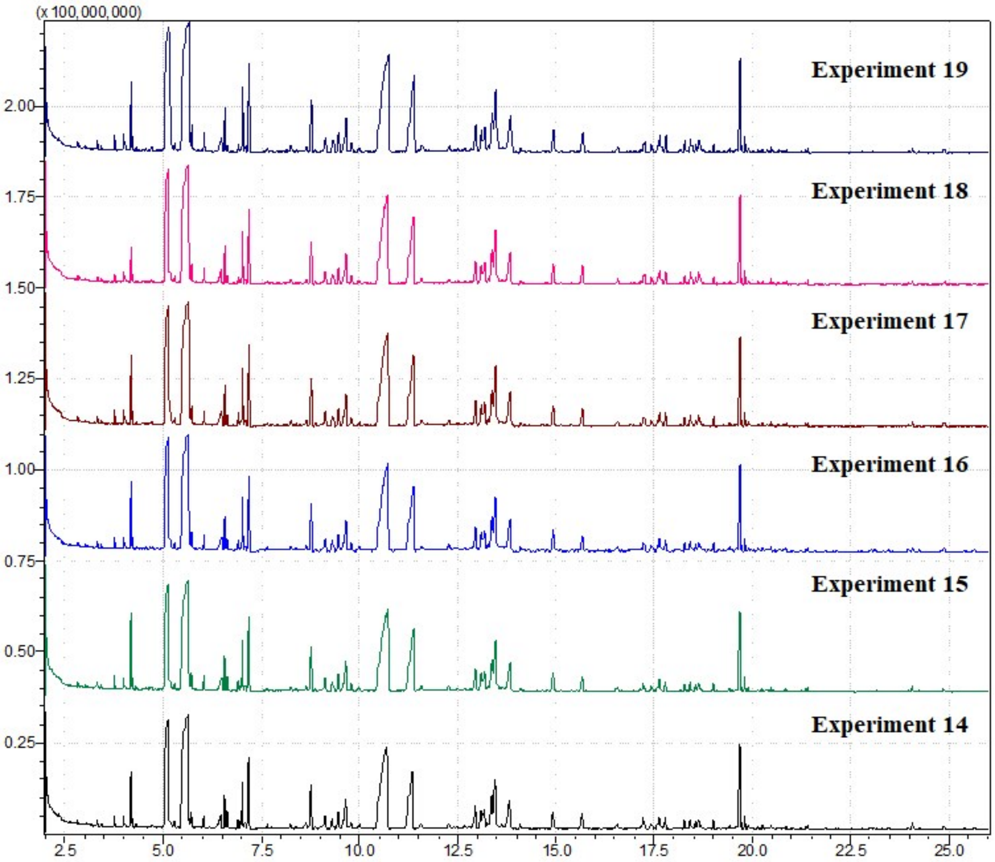
(x 100,000,000)
2.00
1.75
1.50
1.25
1.00
0.75
0.50
0.25
2.5
5.0
7.5
10.0
12.5
15.0
17.5
20.0
22.5
25.0
Experiment 19
Experiment 18
Experiment 17
Experiment 16
Experiment 15
Experiment 14

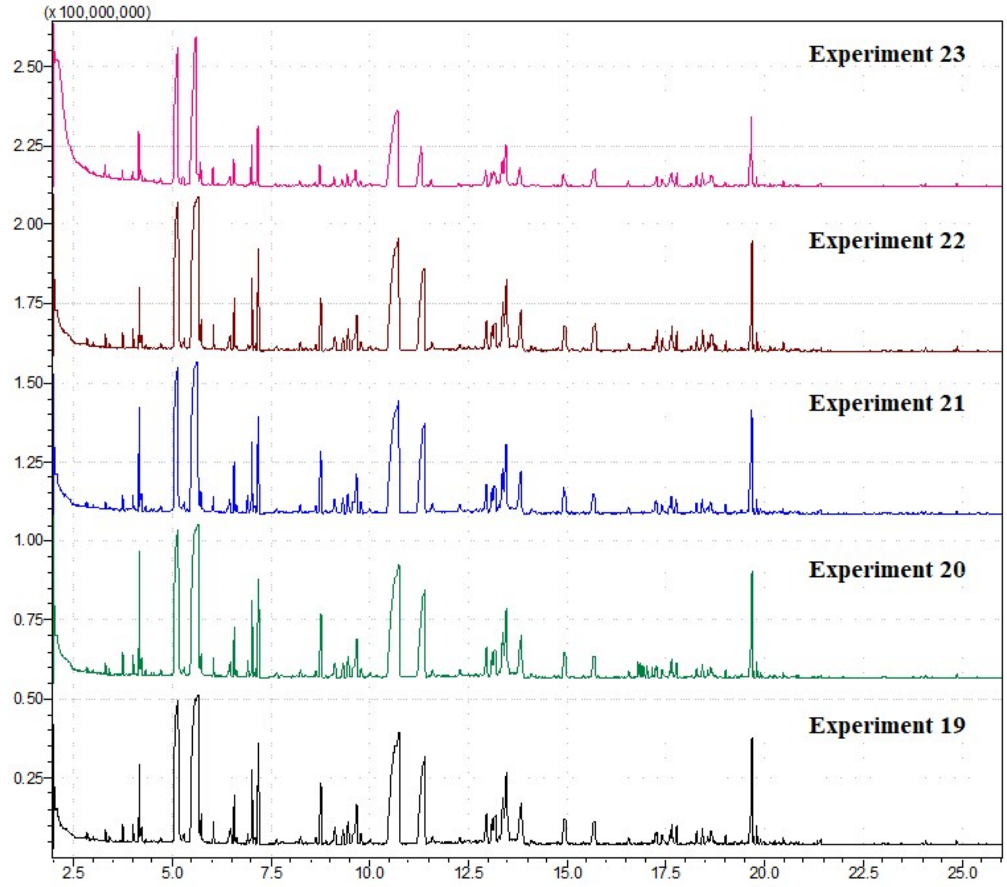
(x 100,000,000)
Experiment 23
Experiment 22
Experiment 21
Experiment 20
Experiment 19
2.50
2.25
2.00
1.75
1.50
1.25
1.00
0.75
0.50
0.25
2.5
5.0
7.5
10.0
12.5
15.0
17.5
20.0
22.5
25.0

3. Chromatograms of the experiments of table 3

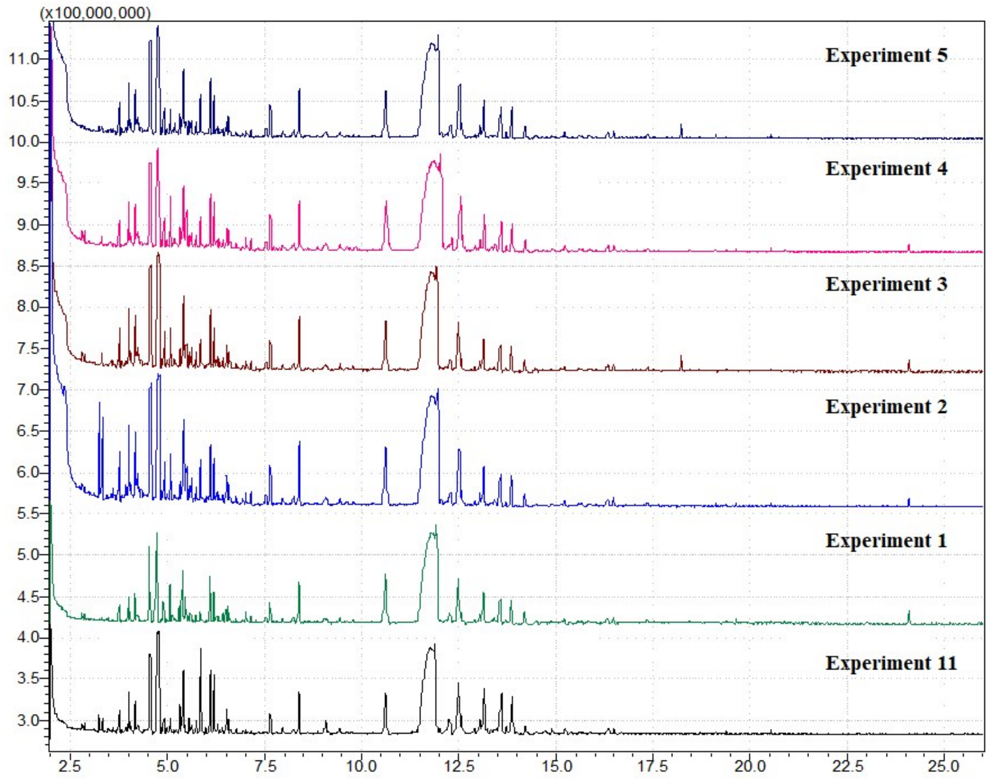

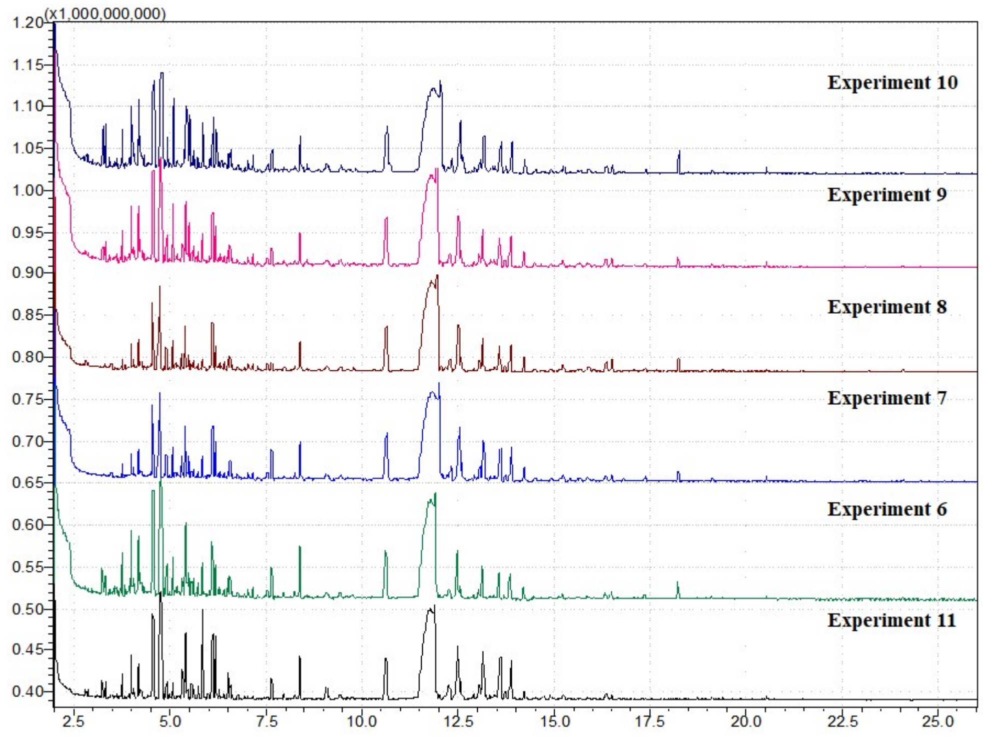

IV. Chemical compositions of the Essentials oils extracted by MAE in the experiments of table 1, 2 and 3.

Table S5: Chemical Composition of essential oil of the Cymbopogom nardus (*Citronella)*

	**Compound**	**% in Essential oil**
1	Citronellal	1,66
2	Citronelol	14,92
3	Geraniol	20,92
4	β-Citral	0,71
5	Citronellyl butyrate	1,12
6	3-Allyl-2-methoxyphenol	0,45
7	3,7-Dimethyl-2,6-octadien-1-ol-acetate	1,9
8	β-elemene	2,98
9	D-Germacrene	2,45
10	4-epi-cubedol	0,85
11	β-elemeno	0,67
12	Germacrene D	0,83
13	(+)-delta-Cadinene	2,27
14	Elemol	19,22
15	Germacren D-4-ol	9,44
16	gama-Eudesmol	1,34
17	delta-Cedrol	1,04
18	α-Cadinol	1,07
19	β-Eudesmol	2,24
20	Ledol	3,8
21	Elemol	3,05
22	Farnesol	1,16
23	1-(4-Hydroxy-7-isopropyl-4-methyloctahydro-1H-inden-1-yl)ethanone	0,89
24	(2E,6E)-2,6-Dimethyl-2,6-octadiene-1,8-diol	0,5
25	7-Hydroxyfarnesen	0,73
26	Elemol	0,56
27	[2-Methyl-2-(4-methyl-3-pentenyl)cyclopropyl]methanol	3,23
	Total	100

Table S6: Chemical Composition of essential oil of *Eucalyptus sp.*

	**Compound**	**% Essential Oil**
1	β Linalol	0,89
2	2-Methylene cyclopentanol	1,62
3	(1R,2R,3R,5S)-(-)-Isopinocampheol	0,79
4	(1R,2R,3R,5S)-(-)-Isopinocampheol	1,85
5	(E)-3(10)-Caren-4-ol	0,69
6	Phellandral	0,53
7	4-Terpineol	5
8	1-Hydrindanone	10,6
9	cis-Piperitol	0,92
10	Longipinene epoxide	0,99
11	5-Ethyl-3-hepten-2-one	0,65
12	p-Cumenol	0,84
13	Cumaldehyde	3,95
14	Phellandral	1,26
15	Cuminol	2,13
16	2-Ethyl-4,5-dimethylphenol	1,21
17	1,7-Dimethylbicyclo[5.2.0] nonane	0,62
18	Isocaryophyllene	1,31
19	Aromadendreno	2,6
20	oxide de Caryophyllene	4,05
21	oxide de Caryophyllene	34,39
22	Ledol	8,54
23	1,2- epoxide Humulene	4,18
24	oxide-(II) Ledene	0,86
25	4,4-dimethyl-Tetracyclo[6.3.2.0(2,5) 0(1,8)] tridecan-9-ol	0,72
26	Isoaromadendrene epoxide	2,03
27	Isoaromadendrene epoxide	2,05
28	Isoaromadendrene epoxide	1,83
29	1b,5,5,6a-Tetramethyloctahydro-6H-indeno[1,2-b]oxiren-6-one	0,81
	Total	97,91

Table S7: Chemical Composition of essential oil of the *Piper aduncum*

	Compound	% Essential Oil
1	Hept-6-en-3-ol	0,83
2	Hept-6-en-3-ol	0,71
3	β-Linalool	42,64
4	Longipinene epoxide	1,45
5	1-Hydroxy-2-pentanone	0,58
6	Hept-6-en-3-ol	0,3
7	Hept-3-enol	0,53
8	cis-Hex-3-enal	0,45
9	trans-Cinnamaldehyde	4,3
10	(Z,Z)-α-Farnesene	1,17
11	(Z,Z)- α-Farnesene	4,11
12	(2E,5E)-2,5-Octadiene	0,64
13	Longipinene epoxide	1,22
14	β-Myrcene	2,68
15	2-[4-(1-Methyl-2-propenyl)phenyl]propanal	1,94
16	Longipinene epoxide	1,06
17	Longipinene epoxide	0,79
18	Longipinene epoxide	1,65
19	Longipinene epoxide	17,36
20	Longipinene epoxide	2,68
21	Methyl 3,6-octadecadiynoate	11,77
	Total	98,86

Table S8: Chemical Composition of essential oil of the *Piper hispidinervum*

	Compound	% Essential Oil
1	1-[(5Z)-5-(1-Hydroxyethylidene)-1,3-cyclopentadien-1-yl]ethanone	1,67
2	Safrole	84,65
3	1-Nonyne	0,46
4	2,4,6-Trimethyl-1-nonene	5,97
5	Longipinene epoxide	6,07
6	p-Menth-8-en-2-ol, acetate	0,48
7	Ledol	0,31
8	Isopropyl decanoate	0,39
	Total	100
